# The prevalence of Lewy body pathology across nine international community-based cohorts

**DOI:** 10.1093/brain/awag112

**Published:** 2026-11-04

**Authors:** Lucy L. Gibson, Claudia K. Suemoto, Johannes Attems, Erin L. Abner, Maria M. Corrada, Laura Gomez-Isaza, Lea T. Grinberg, Alifiya Kapasi, Gregory A. Jicha, Claudia Kawas, C. Dirk Keene, Gabor G Kovacs, Caitlin S Latimer, Renata E. P. Leite, Thomas Montine, Liisa Myllykangas, Allison M. Neltner, Juan Troncoso, Keenan A Walker, Rod Walker, Shih-Hsiu J. Wang, Dag Aarsland, Peter T. Nelson

**Affiliations:** 1 King’s College London, Institute of Psychiatry, Psychology and Neuroscience, London, SE5 9RS, UK; 2 South London and Maudsley NHS Foundation Trust, London, SE5 8AZ, UK; 3 University of Sao Paulo Medical School, Sao Paulo, 05508-220, Brazil; 4 Newcastle University, Newcastle upon Tyne, NE2 4HH, UK; 5 Sanders-Brown Center on Aging, University of Kentucky, Lexington, KY 40536, USA; 6 University of California, Irvine School of Medicine, Irvine, CA 92697, USA; 7 Johns Hopkins School of Medicine, Baltimore, MD 21205, USA; 8 Mayo Clinic, Jacksonville, FL 32224, USA; 9 Rush University Medical Center, Chicago, IL 60612, USA; 10 University of Washington School of Medicine, Seattle, WA 98195, USA; 11 Institute of Neurology, Medical University of Vienna, Vienna 1090, Austria; 12 Tanz Centre for Research in Neurodegenerative Disease, University of Toronto, Toronto, ON M5T 2S8, Canada; 13 Department of Laboratory Medicine and Pathobiology, University of Toronto, Toronto, ON M5S 1A8, Canada; 14 Laboratory Medicine Program, University Health Network, Toronto, ON M5G 2C4, Canada; 15 University of Helsinki and Helsinki University Hospital, Helsinki 00029 HUS, Finland; 16 Laboratory of Behavioral Neuroscience, National Institute on Aging, Baltimore, MD 21224, USA; 17 Kaiser Permanente Washington Health Research Institute, Seattle, WA 98101, USA; 18 Duke University, Durham, NC 27710, USA; 19 Centre for Age-Related Medicine, Stavanger University Hospital, Stavanger 4068, Norway

**Keywords:** Lewy body pathology, neuropathology, dementia with Lewy bodies

## Abstract

Reported prevalence estimates of Lewy body pathology (LBP) vary widely, often without considering brain regional distributions or demographic influences. Large, population-representative autopsy cohorts are needed to provide estimates and clarify the distribution and clinical implications of LBP.

Neuropathological, genetic, and clinical data were pooled from nine community- or population-based brain autopsy cohorts in the USA (n=6), Brazil, Austria, and Finland (n=7309 total; 59% women; mean age of death 84.2 years). Cognitive status was available for 6166 participants: dementia (38.5%), mild cognitive impairment (14.8%), and cognitively unimpaired (47.0%). Frequency of LBP was examined by anatomical distribution (neocortical, limbic, brainstem, amygdala, olfactory) and stratified by covariates. Prevalence was calculated in meta-analysis, and mixed-effect logistic regression examined associations with sex, cognitive status, Alzheimer’s disease neuropathological change (ADNC), and limbic-predominant age-related TDP-43 encephalopathy neuropathologic change (LATE-NC) co-pathology.

Overall, 27.3% of participants had LBP: neocortical 8.4%, limbic 6.8%, brainstem 4.8%, amygdala 3.7%, and olfactory 3.6%. Neocortical LBP was associated with dementia (present in 15%; OR=4.06 [95%CI 3.24-5.10]) and with increased odds of ADNC (OR=2.30 [95%CI 1.88–2.81]) and LATE-NC (OR=2.03 [95%CI 1.69-2.44]). Sex differences were not observed in the frequencies of neocortical or limbic LBP. However, amygdala-predominant LBP was more frequent in women (OR=1.53 [95%CI 1.06-2.21]) whereas brainstem-predominant LBP was more common in men (OR=0.64 [95%CI 0.49-0.84]). Additionally, amygdala-predominant LBP was associated with increased odds of comorbid ADNC (OR=12.7 [95%CI 5.30–30.6]) while brainstem-predominant LBP was not associated with greater odds of ADNC co-pathology (OR=0.88 [95%CI 0.67–1.16]).

Pooling data from large, international community-based autopsy cohorts allows for more robust estimates of region-specific LBP prevalence and their associations with cognitive status. The observed sex- and co-pathology–specific differences in brainstem and amygdala-predominant LBP highlight potential biological heterogeneity and suggest that distinct disease pathways may underlie these patterns.

## Introduction

Lewy body pathology (LBP) is characterized by the accumulation of intracellular α-synuclein fibrils forming Lewy bodies (LB) and Lewy neurites. These pathologic hallmarks are enriched in a spectrum of clinically defined conditions, including Parkinson’s disease (PD), Parkinson’s disease dementia (PDD) and dementia with Lewy bodies (DLB), the latter two often combined as Lewy body dementia (LBD). Prior studies have reported wide variations in the prevalence of LBD. In a systematic review of community-based cohorts, DLB accounted for 4.2% of dementia cases,^1^ but some studies have reported prevalence estimates of up to 16%.^2^ However, these estimates are largely based on clinical diagnoses without neuropathological confirmation, and LBD is believed to be substantially underdiagnosed, with frequent misclassification and diagnostic delay.^3,4^ Presumed preclinical LBP, often of lower severity, is also found in some individuals without clinical symptoms, and may be referred to as “incidental Lewy body disease”.^4–7^ The reported population prevalence of LBP varies widely, ranging from 6-61%, reflecting heterogeneity in study design, neuropathological assessment protocols, and the demographic and cognitive characteristics of the studied populations. ^8–14^

Several different criteria have been proposed for the staging of LBP. The original Braak PD pathologic grading model hypothesised a six-stage caudo-rostral progression of LBP from the brainstem (medulla oblongata) to neocortex,^15^ whereas more recent consensus criteria classify LBP by its predominant anatomical distribution (neocortical, limbic, brainstem, amygdala, or olfactory only).^16,17^ Of the LBP subtypes, neocortical and limbic-predominant LBP are more often associated with dementia, with prior neuropathological studies attributing 15–20% of dementia cases to LBP, while brainstem-predominant LBP is the main substrate for clinical PD and differing results have been reported for the clinical correlates of amygdala-predominant LBP.^13,17–21^ Moreover, most prior prevalence studies have not reported the regional distribution of LBP, limiting insight into the clinical correlates of these subtypes.^22^

Many existing prevalence estimates of LBP derive from clinic-based studies, which overrepresent atypical or severe phenotypes and underrepresent mixed pathologies, limiting their generalisability.^23^ Larger, population-representative and community-based cohorts are needed to provide relatively unbiased estimates of prevalence and to clarify the frequency, anatomical distribution, and clinical correlates of LBP.^24^ Furthermore, the associations between LBP and basic demographic parameters also remain unclear. Although several studies report higher rates of LBP with advancing age,^9,14,25^ others have not found an association with age.^11,20,26^ Similarly, sex differences are inconsistent across studies; while clinical DLB and PDD cohorts typically have more men, findings from population-based and neuropathological studies are mixed.^27–32^

LBP rarely occurs in isolation and neuropathological heterogeneity is common.^33^ Depending on the criteria being used, up to 50% (or even more) of cases with LBP have concurrent Alzheimer’s disease neuropathological change (ADNC).^34^ Other co-pathologies including limbic age-related TDP-43 encephalopathy neuropathological change (LATE-NC) and cerebrovascular disease (CVD) are also common and increase in frequency with age.^19,35–43^ The coexistence of LBP and ADNC in particular is associated with accelerated cognitive decline, greater burden of neuropsychiatric symptoms, and lower clinical diagnostic accuracy than either pathology alone.^34,44–50^ It is not clear how the joint prevalence of co-pathologies may differ with the distribution of LBP, but prior clinical studies demonstrate higher prevalence of co-morbid ADNC in DLB than in PD.^37^ Other studies have suggested increased ADNC co-pathology in amygdala-predominant LBP,^51^ possibly reflecting a greater spread of ADNC in ‘brain first’ spread of LBP, but this needs further characterisation.^52,53^

The true prevalence of LBP remains unknown and represents a major unmet need for improving early detection, diagnostic accuracy, and therapeutic trial design. Robust prevalence estimates from large, geographically diverse, community- and population-based autopsy cohorts are needed to better define the population burden of LBP and its associated risk factors. Although previous reports from single cohorts and consortia have provided important insights,^22^ no prior study has combined data from multiple community-based cohorts to examine the prevalence of LBP by anatomical distribution and explore associations with co-pathology, demographic and cognitive characteristics. Addressing this need, we examined the prevalence and regional distribution of LBP in nine well-characterized, community-based autopsy cohorts spanning North America (6), South America (1), and Europe (2). By pooling data across cohorts, we aimed to determine how frequently LBP was observed post-mortem, its associations with age, sex and cognitive status and how clinical and pathological covariates differed across regional LBP distributions.

## Materials and methods

### Cohorts and data sources

Summary data were collected for participants with neuropathological assessment of LBP from nine well characterised community- or population-based brain aging cohorts: the Adult Changes in Thought (ACT) study^54^; Baltimore Longitudinal Study of Aging (BLSA)^55,56^; Biobank for Aging Studies from the University of Sao Paulo (BAS-USP)^57–59^; Duke University/University of North Carolina Alzheimer’s Disease Research Center (Duke/UNC-ADRC)^60^; Rush University Religious Orders Study/Memory and Aging Project (ROS-MAP)^61,62^; University of California, Irvine 90+ Study ^63,64^; University of Kentucky Alzheimer’s Disease Research Center (UKy-ADRC)^65,66^; Vantaa 85+ Study^67^; and Vienna Trans-Danube Aging (VITA) Study^12,68,69^. Data from eight of these cohorts has been published previously as a consortium, but data on LBP was not reported.^70^

Characteristics of these cohorts are summarized in Supplementary Table 1, with additional specific cohort details provided in Supplementary Table 2. Each study obtained approval from its institutional review board or ethics committee, and participants (or next of kin) provided informed consent. Most analyses were performed on de-identified summary data (i.e. cohort-level data) and no additional ethical approval was required.

### Population characteristics

Several included cohorts were population-based, recruiting from defined geographic areas with the aim of sampling broadly from the underlying population. Others were large community-based convenience cohorts, often recruiting through community outreach activities such as media advertisements, health fairs, and local presentations. While lacking the unbiased sampling of population-based studies, these cohorts are generally more representative than clinic- or hospital-based series, given their size and the absence of pre-selection into clinicopathological groups. Additional details regarding recruitment are provided in Supplementary Table 2.

### Neuropathological assessment

Structured data requests for standardised neuropathological measures were sent to each cohort. LBP was staged in all participants: eight cohorts used the McKeith criteria (classifying neocortical-, limbic-, brainstem-, or amygdala-predominant LBP),^16^ and one cohort (UCI90) applied only the Braak PD staging (0–VI).^15^ Three cohorts also reported ‘olfactory-only’ LBP in line with updated consensus recommendations^17^ (Supplementary Table 1). The ROS–MAP cohort used an earlier modified version of the McKeith classification including only neocortical-, limbic-, and brainstem-predominant categories.^71,72^ All participating sites performed α-synuclein immunohistochemistry, with site-specific antibody technical details described in Supplementary Table 1. There was some variation in tissue processing, neuropathological methods, and data completeness across cohorts, described in Supplementary Table 2.

Additional requested measures from each cohort included Braak neurofibrillary tangle (NFT) staging (0–VI scale)^73^ performed using anti-phospho-Tau antibodies; the Consortium to Establish a Registry for Alzheimer’s Disease (CERAD) neuritic amyloid plaque density scores (graded as “None”, “Sparse plaques”, “Moderate plaques”, or “Frequent plaques”)^74^ and Thal Aβ phases (a 0–5 scale based on anatomic distribution of Aβ plaques detected with Aβ immunostaining).^75^ Overall ADNC severity was classified for each case according to NIA-AA criteria, based on combined Thal Aβ phase, Braak NFT stage, and CERAD neuritic plaque score.^76^ TDP-43 proteinopathy distribution provided the bases of LATE-NC staging, all cohorts sampled amygdala, hippocampus, and middle frontal gyrus.^77^

### Clinical assessment

Summary data for sex, age, *APOE ε*4 status, cognitive status at final assessment prior to death (dementia, mild cognitive impairment [MCI], cognitively unimpaired [CU]) were also provided. Cognitive status data was available from seven cohorts, primarily through clinical evaluations in life with diagnoses made via consensus conference, except in the BAS, where status was determined post-mortem from validated next-of-kin interviews.^78,79^ Diagnostic criteria for dementia varied across studies (detailed in Supplementary Table 1); for example, the VANTAA 85+ cohort used DSM-III-R criteria to identify dementia and did not classify MCI and in the UCI 90+ study MCI was classified according to the cognitive impairment, no dementia (CIND) criteria which is less stringent than MCI classification.^80^

Three cohorts (Duke/UNC-ADRC, UKy-ADRC and UCI90+) identified a subgroup of participants who had clinical diagnoses of PDD or DLB made during life. Four cohorts also provided data on core clinical features of DLB, but assessment methods of these symptoms were not standardized; BAS-USP relied on informant ratings from the Tanner scale for parkinsonism and Neuropsychiatric Inventory items for sleep and hallucinations, whereas participants were clinically assessed in other cohorts.

### Statistical analyses

Random-effects meta-analyses were performed using restricted maximum likelihood estimation to account for between-cohort heterogeneity for each regional subtype of LBP. Study-specific proportions were pooled using inverse-variance weighting and are presented as pooled proportions with 95% confidence intervals. For the purposes of the analyses the Braak PD staging was approximated to neocortical (stage 5-6), limbic (stage 3-4) and brainstem (1-2) distributions.

Joint distributions of co-pathologies (Braak NFT stage, Thal Aβ phase, CERAD neuritic plaque score, and LATE-NC stage) were calculated for each anatomical subtype of LBP (neocortical, limbic-, brainstem- and amygdala-predominant) summed across the cohorts. Olfactory-only was not included in this analysis because it was not reported across a sufficient number of cohorts. ADNC was defined according to recent consensus recommendations, using combined Thal (Aβ), Braak (NFT), and CERAD (neuritic plaques) criteria to generate overall prevalence estimates.^81^ Demographic characteristics, including mean age at death, sex distribution, and *APOE* ε4 frequency, were summarized as cohort-specific estimates weighted by sample size. Univariable meta-regression analyses examined associations between the prevalence of LBP subtypes with mean age at death or *APOE ε*4, and correlations were explored with Spearman’s correlation. The VITA and BLSA cohorts were excluded from APOE analyses because genotype data were not available. To explore other potential sources of heterogeneity, subgroup analyses and meta-regression examined differences by study design (population-based or community-based cohorts) and geographic location (USA versus non-USA cohorts).

Prevalence estimates of LBP by regional distribution were aggregated across cohorts and stratified by cognitive status and sex, with differences across groups evaluated with χ^2^ tests. Overall prevalence and between-cohort heterogeneity were further examined using random-effects meta-analysis Associations between LBP distribution and cognition or sex were assessed using mixed-effects logistic regression models for each LBP region, adjusted for mean age at death and sex distribution, with a random intercept for cohort with results presented as odds ratios (OR). Similar models estimated the odds of ADNC and LATE-NC across LBP regions. Statistical significance was assessed at the 5% level with Bonferroni correction applied to these models. Variability in Braak NFT staging across cohorts was examined in meta-analysis to identify sources of heterogeneity for each LBP subtype. Furthermore, to examine whether co-pathology contributed to sex differences in LBP, subgroup descriptive analyses were performed across three cohorts which provided data stratifying co-pathology across different LBP distributions by sex and cognitive status. Multinomial logistic regression, adjusted for the individual sex of participants and cohort, was used to examine associations between sex, cognitive status and both ADNC and LATE-NC with results presented as relative risk ratios (RRR).

Clinical data were summarised across three cohorts (Duke/UNC-ADRC, UKy-ADRC and UCI90) to describe the distribution of LBP subtypes in participants with clinical diagnoses of PDD or DLB. Participants from cohorts were only included in this analysis if clinical diagnoses of PDD or DLB were made during life. Additionally, the distribution of core clinical features among participants with each anatomical subtype of LBP was described. All statistical analyses were performed in Stata/SE version 18.5 (StataCorp).

## Results

Across the nine cohorts there was a total of 7309 participants included in the current study. The numbers of participants contributing to the data from each cohort are described in Table 1 along with demographic, clinical, genetic and summary neuropathologic data. The median number of included participants per cohort was 356 (range 264-2208). The mean weighted age at death across cohorts was 84.2 years (range of mean 72.9-97.2) and 59% of included participants were women. The APOE genotype was known in 5655 participants and 27.5% of these participants had an *APOE ε*4 allele. The cognitive status was reported in seven cohorts for a total of 6616 participants; 47.0% were classified as CU, 14.8% had a diagnosis of MCI and 38.5% were diagnosed with dementia at their last clinic visit before death.

### Prevalence of LBP

Meta-analysis of the overall frequency of LBP across the community-based cohorts separated by regional distribution is shown in Figure 1. Across the whole sample 27.3% of participants had LBP: neocortical LBP was reported in 8.4% of participants (95% CI 5.2-11.7%); limbic-predominant LBP was reported in 6.8% (95% CI 4.3-9.3%); and brainstem-predominant LBP was reported in 4.8% (95% CI 2.8-6.8%). The prevalence of amygdala-predominant LBP was 3.7% across 7 cohorts (95% CI 1.9-5.6%) and olfactory only LBP was identified in 3.6% (95% CI 0.5-6.7%) of participants in the 3 cohorts reporting this subtype. There was heterogeneity across cohorts in the prevalence of LBP, ranging from 2.1-14.4% for neocortical LBP, 1.7-12.9% for limbic-predominant LBP, 1.8-10.2% brainstem-predominant LBP, 0.8-7.1% amygdala-predominant LBP and 0.9-5.4% for olfactory only LBP (see Figure 1).

### Prevalence of LBP by cognitive status

Analyses were conducted using data from the seven cohorts that reported cognitive status. For each LBP subtype, meta-analyses were performed stratified by cognitive status across the community-based cohorts. The overall prevalence and 95% confidence intervals are presented in Table 2, with corresponding meta-analyses shown in Supplementary Figures 1–4.

Among participants with dementia, 15% had neocortical LBP, 8% limbic-predominant LBP, 4% brainstem-predominant LBP, 6% amygdala-predominant LBP and 3% olfactory-only LBP. In the cohorts reporting MCI, 8% of participants had neocortical LBP, 4% limbic-predominant, 5% brainstem-predominant, 2% amygdala-predominant and 3% olfactory only LBP. Low percentages of LBP were reported in CU individuals (4% neocortical LBP, 6% limbic-predominant, 5% brainstem-predominant, 1% amygdala-predominant and 2% olfactory only LBP). Heterogeneity in the prevalence of LBP was observed across cohorts even when stratified by cognitive status. Neocortical LBP ranged from 6-21% in dementia, 2-11% in MCI and 1-9% in CU participants (Supplementary figure 1). Limbic-predominant LBP ranged from 2-16% in dementia, 1-8% in MCI and 2-13% in CU participants (Supplementary figure 2).

Associations between LBP subtypes and cognitive status were examined in mixed effect logistic regression with cohort included as a random intercept and adjusted for the overall sex distribution and mean age of the cohort (Figure 2). Neocortical LBP was more frequent in participants with dementia (OR 4.02, 95% CI 3.20–5.05; p < 0.001) and MCI (OR 1.79, 95% CI 1.31–2.45; p < 0.001) compared with CU individuals, and was also more frequent in dementia than MCI (OR 2.24, 95% CI 1.72–2.91; p < 0.001).

Limbic LBP showed modest increases in dementia compared with CU (OR 1.30, 95% CI 1.03–1.65; p = 0.027) and MCI (OR 1.49, 95% CI 1.06–2.08; p = 0.021), but these associations did not remain significant after Bonferroni correction. Limbic LBP did not differ between MCI and CU (OR 0.88, 95% CI 0.62–1.25; p = 0.462). Brainstem LBP was not associated with cognitive status. Although brainstem LBP was less frequent in dementia than CU (OR 0.70, 95% CI 0.51–0.97; p = 0.030), this did not survive correction for multiple comparisons. Amygdala-predominant LBP was more frequent in dementia than CU (OR 4.99, 95% CI 3.08–8.11; p < 0.001) and MCI (OR 2.71, 95% CI 1.45–5.42; p = 0.023); only the comparison with CU remained significant after Bonferroni correction. Amygdala-predominant LBP did not differ between MCI and CU (OR 1.84, 95% CI 0.72–4.71; p = 0.20).

### Association between LBP and co-pathologies

Co-pathologies across all participants with each anatomical subtype of LBP are described in Table 3. Severe burden of NFTs (Braak NFT stages V-VI) was more frequent in neocortical LBP (42.2%) compared with limbic- (33.7%) and brainstem-predominant LBP (16.1%). Similarly, frequent CERAD neuritic plaques were more common in neocortical (38.8%) than limbic (30.6%) or brainstem-predominant LBP (20.9%). Advanced Thal Aβ phases (4–5) were present in 57.1% of neocortical, 46.3% of limbic, and 34.2% of brainstem-predominant LBP cases.

Fewer cohorts staged amygdala-predominant or olfactory-only LBP, as reflected by the smaller sample size. However, the prevalence of ADNC co-pathology in these groups was markedly higher than in the whole cohort: among amygdala-predominant cases, 73.3% had severe NFTs, 46.0% had frequent CERAD plaques, and 80.9% had Thal Aβ phase 4–5.

### ADNC and LBP

Among participants without LBP, ADNC was present in 45.0% (n = 2471). In those with LBP, the prevalence varied by anatomical subtype of LBP: it was highest in neocortical LBP (74.3%, n = 442), followed by limbic-predominant LBP (61.1%, n = 247), and brainstem-predominant LBP (47.7%, n = 114). In three cohorts reporting ADNC in amygdala-predominant LBP, ADNC was observed in 88.7% (n = 47) of cases.

Mixed-effects logistic regression models with cohort included as a random intercept and adjusted for the overall sex distribution and mean age of the cohort showed that neocortical LBP was associated with more than twofold higher odds of ADNC compared with individuals without neocortical LBP (OR 2.30, 95% CI 1.88–2.81; p < 0.001) (Figure 3). Limbic-predominant LBP was also significantly associated with ADNC, though to a lesser extent (OR 1.40, 95% CI 1.13–1.74; p = 0.002). However, brainstem-predominant LBP was not significantly associated with ADNC (OR 0.88, 95% CI 0.67–1.17; p = 0.386). Amygdala-predominant LBP showed a strong association with ADNC in the three cohorts reporting this subtype, albeit with wider confidence intervals due to smaller numbers (OR 12.74, 95% CI 5.30–30.58; p < 0.001). Between-cohort variance was modest (intraclass correlation coefficient 0.028, 95% CI 0.010–0.079).

To explore variability in the distribution of Braak NFT co-pathology across studies, a random effects meta-analysis was conducted for each regional subgroup of LBP shown in supplementary figures 5–8. As expected, there was substantial heterogeneity in the prevalence of NFT across groups but the pooled, weighted estimate generated by random effects models was similar to the prevalence estimated from total numbers.

### LATE-NC and LBP

LATE-NC was also most frequent in neocortical LBP (42.3%), followed by limbic (29.3%) and brainstem LBP (22.4%). In mixed-effects logistic regression models adjusted for age and sex and including cohort as a random intercept shown in Figure 3, the odds of LATE-NC were increased in individuals with neocortical LBP (OR 2.03, 95% CI 1.69–2.44; p < 0.001) and amygdala-predominant LBP (OR 2.15, 95% CI 1.46–3.18; p < 0.001). Limbic-predominant LBP was associated with an increase in odds of LATE-NC (OR 1.27, 95% CI 1.00–1.61; p = 0.047) but this did not meet the statistical significance threshold after Bonferroni correction. Brainstem-predominant LBP was not associated with LATE-NC (OR 1.04, 95% CI 0.75–1.44; p = 0.831).

### Cognitive status and co-pathology

Co-pathology in LBP stratified by cognitive status and sex distribution was available in 3 cohorts (BAS-USP n=2075, ROS-MAP n=2087, UKy n=217). Across all anatomical subtypes of LBP, co-existing ADNC and LATE-NC were significantly more frequent in participants with dementia (Table 4).

In multinomial logistic regression adjusted for the cohort and individual sex of participants with neocortical LBP, comorbid ADNC was associated with an increased relative risk of dementia compared to CU participants (RRR=5.53 [95% CI 2.98–10.2], p < 0.001) and MCI (RRR=3.92 [95% CI 2.06–7.47]; p<0.001). There was no significant difference in risk with co-morbid ADNC between MCI and CU (RRR=1.41[95% CI0·66–2.99]; p=0.372). Similarly, co-morbid **LATE-NC** in neocortical LBP was associated with an increased relative risk of dementia versus CU (RRR= 3.00 [95% CI 1.59–5.68]; p=0.001), but not versus MCI (RRR=1.73 [95% CI 0.93–3.21]; p=0.085) or between MCI and CU (RRR=1.74 [95% CI 0.77–3.93]; p=0.182).

Similarly, in participants with limbic LBP, comorbid ADNC was associated with an increased relative risk of dementia versus CU (RRR=6.81 [95% CI 3.41-13.6], p < 0.001) and MCI (RRR=3.74 [95% CI 1.69–8.23], p = 0.001) but there was no significant difference in risk between MCI and CU with comorbid ADNC (RRR=1.82 [95% CI 0.79–4.18]; p = 0.157). Comorbid LATE-NC in limbic LBP was also associated with an increased relative risk of dementia versus CU (RRR=6.81 [95% CI 3.41-13.6], p < 0.001) and versus MCI (RRR=2.76 [95% CI 1.22–6.24], p = 0.015), but not with MCI versus CU participants.

In participants with **brainstem LBP**, co-morbid ADNC was not associated with increased risk of dementia or MCI. Co-morbid LATE-NC was associated with greater relative risk of dementia compared with CU (RRR=8.03 [95% CI 1.94-33.2], p = 0.004), but there was no significant difference in risk between dementia and MCI or between MCI and CU. Multinomial regression could not be performed for the **amygdala-predominant LBP** group because of small sample size; however, it is notable that all participants with dementia and amygdala-predominant LBP had co-morbid ADNC (Fisher’s exact p<0.001).

### Association with demographic and genetic factors

#### Age of death

The mean age at death was positively associated with the frequency of neocortical LBP (r = 0.79, *p*=0.01), but no significant correlations were observed for limbic, brainstem, or amygdala-predominant LBP (Supplementary figure 9). Consistent with this, meta-regression showed that age at death was significantly associated with the proportion of neocortical LBP (β=0.01 [0.002–0.01], *p*=0.001) but not with LBP in other regions (limbic β=0.002 [−0.002–0.005], *p*=0.44; brainstem β=0.002 [−0.001–0.005], *p*=0.15; amygdala β=0.001 [−0.002–0.003], *p*=0.37).

#### *APOE ε*4 genotype status

There was no correlation between the cohort percentage with *APOE* ε4 positivity and the proportion of the reported subtypes of LBP as shown in supplementary figure 10.

#### Epidemiological factors

There was no evidence of geographic variation in regional LBP prevalence between US and non-US cohorts. Neither test of subgroup differences (neocortical Q_(between)_(1) = 0.60, p = 0.44, limbic: Q_(between)_(1) = 0.00, p = 1.00, brainstem Q_(between)_(1) = 0.19, p = 0.67, amygdala Q_(between)_(1) = 0.38, p = 0.54) nor age-adjusted meta-regression (neocortical β = 0.00 [−0.06-0.06], p=0.81; limbic β = −0.19 [−0.09-0.05], p=0.58; brainstem β = −0.03 [−0.07-0.014], p=0.18; amygdala β = 0.02 [−0.02-0.06], p=0.40) showed any association. Similarly, LBP prevalence did not differ between population-based and community-based cohorts in any brain region (neocortical Q(between)(1) = 0.70, p = 0.40, limbic: Q(between)(1) = 0.00, p = 1.00, brainstem: Q(between)(1) = 0.00, p = 0.99, amygdala: Q(between)(1) = 1.67, p = 0.20) and age-adjusted meta-regression demonstrated no association between study design and LBP subtype prevalence (neocortical β = −0.006 [−0.056-0.04], p=0.81; limbic β = 0.008 [−0.05-0.07], p=0.81; brainstem β = 0.01 [−0.032-0.055], p=0.61; amygdala β = −0.01 [−0.05-0.03], p=0.61).

#### Sex

The frequency of neocortical and limbic LBP, each summed across cohorts, did not differ by sex (Table 5). The proportion of each type of LBP was evaluated in meta-analyses, the pooled frequency is reported in table 5 and forest plots are shown in supplementary Figures 11–14. In univariate analyses brainstem-predominant LBP was significantly more common in men (*X*^2^ = 6.34, p = 0.012) and amygdala-predominant LBP was significantly more common in women (*X*^2^ = 9.82, p = 0.002). All other neurodegenerative pathologies staged in the cohorts (amyloid plaque severity stages, Braak NFT stages and LATE-NC stages) were significantly more common in women than men as illustrated in table 5.

In mixed-effects logistic regression models adjusted for age and including cohort as a random intercept, the odds of neocortical and limbic LBP did not differ by sex (supplementary figure 15; neocortical: female vs male OR 0.89, 95% CI 0.74–1.06; p = 0.184; limbic: OR 0.87, 95% CI 0.71–1.07; p = 0.197). In contrast, brainstem-predominant LBP was more frequent in men (female vs male OR 0.64, 95% CI 0.49–0.84; p = 0.001) while amygdala-predominant LBP was more frequent in women than men (female vs male OR 1.53, 95% CI 1.06–2.22; p = 0.023), but this association did not remain significant after Bonferroni correction. Together, these findings suggest sex-related differences in LBP vary across regional distribution.

#### Subgroup analysis for ADNC co-pathology in LBP by sex

Subgroup analysis of three cohorts assessed variation in ADNC co-pathology across regional subtypes of LBP by sex. In these cohorts, a higher proportion of women with neocortical and limbic LBP tended to have ADNC than men but the difference was not statistically significant (Supplementary table 3).

### Concordance with clinical diagnosis and core clinical features

Three of the nine cohorts (Duke/UNC ADRC, UCI90, UKy-ARDC) reported clinical diagnoses made during life. There were 29 clinical diagnoses of DLB and 11 diagnoses of PDD across the cohorts (Duke n=5; UK n=13; UCI90 n=22). Across participants with a clinical diagnosis of DLB, 41.3% had neocortical LBP, 7.9% had limbic-predominant LBP and 17.2% had brainstem-predominant LBP and 34.5% had no LBP (Supplementary table 4). In participants with PDD, 45.5% had neocortical LBP, 36.4% had limbic LBP, 9.1% had brainstem-predominant LBP and 9.1% had no LBP. Limbic LBP was more common in PDD than DLB but there were no other significant differences in neuropathology across the clinical groups.

Three cohorts (UKy-ADRC, Duke/UNC-ADRC and BAS-USP) included additional clinical information for a subgroup of participants with LBP. In participants with neocortical LBP, 38.0% (n=30) reported hallucinations, 60.3% (n=44) reported parkinsonism, 25.0% (n=9) reported cognitive fluctuations and 33.3% (n=26) reported sleep disturbance. Core clinical features were less frequently reported in limbic, brainstem and amygdala-predominant LBP shown in supplementary figure 16.

## Discussion

In this large, international, aggregated sample of 7309 autopsied research participants across 9 population- and community-based cohorts, over a quarter of participants had LBP (neocortical 8%; limbic-predominant 7%; brainstem-predominant 5%; amygdala-predominant 4%; olfactory only 4%). Among participants with dementia, neocortical LBP was most frequent (15%), followed by limbic-predominant (8%), amygdala-predominant (7%) and brainstem-predominant LBP (4%). However, the prevalence of clinically diagnosed LBD in dementia populations has previously been estimated as 5%.^4^ The discrepany with the current post-mortem study significant underdiagnosis in clinical populations, with the majority of individuals with neocortical or limbic LBP not receiving a diagnosis of LBD. Among those with MCI, 8% had neocortical LBP and 4% had limbic LBP, providing a relatively population-generalizable estimate of LBP in MCI populations that exceed prior clinical estimates of prodromal DLB in MCI.^82^

Additionally, the odds of neocortical LBP were more than four times greater in participants with dementia compared with CU individuals and more than twofold higher than those with MCI.^17^ Limbic-predominant LBP was also more common in dementia, although the odds of dementia in this LBP subgroup were lower than neocortical LBP. Presence of co-morbid ADNC and LATE-NC in neocortical and limbic LBP further increased the odds of dementia, highlighting the additive impact of each co-pathology on cognitive decline. In contrast, the prevalence of brainstem-predominant LBP was stable across cognitive groups and showed no association with dementia, supporting an aetiological role for neocortical and limbic, but not brainstem, LBP in cognitive decline. Furthermore, although earlier reports indicate up to 25% of older people without dementia have LBP without clinical manifestations,^17,83^ only 11% of CU participants in the current study had LBP (neocortical 3%, limbic 4%, brainstem 3% and amygdala-predominant LBP 1%) suggesting prior studies may have overestimated incidental LBP. Although the prevalence of amygdala-predominant and olfactory only LBP was higher in dementia groups, this most likely reflects the high burden of concomitant ADNC co-pathology observed in these populations. Indeed, in sub-analyses, all participants with amygdala-predominant LBP and dementia had co-morbid ADNC, indicating that amygdala LBP alone is not a driver of dementia.

The neuropathological heterogeneity of LBP is well established, with ‘pure’ disease relatively uncommon.^84–86^ In the current study, co-pathology was frequent, particularly in neocortical LBP, where 74% of participants had co-morbid substantial ADNC and 42.3% had stage 2 or 3 LATE-NC—higher than prior estimates not stratifying by distribution of LBP.^34^ These findings suggest that synergistic interactions between ADNC, LATE-NC, and LBP may be particularly important in neocortical LBP.^87,88^ The odds of ADNC and LATE-NC were also markedly elevated in amygdala-predominant LBP, where severe NFT and diffuse amyloid pathology were common, supporting earlier reports of a link between amygdala involvement and both ADNC and LATE-NC.^51,89,90^ In contrast, the odds of ADNC co-pathology were not increased in brainstem-predominant LBP. Recent evidence suggests two possible trajectories of LBP progression: a caudo-rostral ‘body-first’ pathway and an amygdala- or olfactory ‘brain-first’ pathway.^52,53^ The high prevalence of ADNC reported in amygdala-predominant relative to brainstem-predominant LBP suggests that comorbid ADNC may be more characteristic of the ‘brain-first’ variant, contributing to clinical heterogeneity in LBD. There may also be protective factors inherent to brainstem-predominant LBP that limit the spread of multiple pathologies to supratentorial regions.

Previous studies have reported varying results on the association between age and prevalence of LBP, with some reporting stable prevalence across the ageing spectrum (or even decreased prevalence in persons living beyond 90 years of age), while others describe increasing prevalence with age.^8,25^ In the present study, cohort-level increased age was associated with increased prevalence of neocortical, but not other distributions of LBP which may help explain inconsistencies across earlier reports not differentiating by anatomical subtype of LBP.

Although clinical cohorts of DLB and PDD typically report higher prevalence in men,^28,34^ in the current study we found no sex differences in the prevalence of neocortical or limbic LBP—the subtypes most associated with dementia—suggesting that women with LBP may be underdiagnosed in these clinical cohorts. Indeed, prior studies suggest men more often exhibit parkinsonism and REM sleep behavioural disorder, clinical features that facilitate diagnosis and increase representation in specialty clinics,^36,91^ while women often present with fewer core features, contributing to higher rates of misdiagnosis and diagnostic delay.^29^ Co-pathology may also influence presentation: pure neocortical LBP is more common in men, whereas women more often have concomitant ADNC in mixed disease presentations which are also associated with fewer core features and higher rates of misdiagnosis.^19,27,32,92–95^

In the current study, other neurodegenerative pathologies were more common in women, however, analysis of ADNC across LBP subtypes by sex was limited by practical factors to a subgroup of three cohorts and did not reach statistical significance, likely due to low statistical power.

However, other sex-related differences in the regional distributions of LBP were observed with higher odds of brainstem-predominant LBP in men and the greater prevalence amygdala-predominant LBP in women. These findings suggest that men and women may be differentially affected by pathological mechanisms, with brainstem-associated ‘body-first’ subtypes more common in men and amygdala-onset ‘brain-first’ subtypes more common in women.^96^ The differences in the prevalence of ADNC across amygdala and brainstem-predominant distributions, with ADNC also more common in women than men suggests there may be common factors driving these pathways. Such divergence may contribute to sex-specific patterns of clinical manifestation and underdiagnosis. Recognition of these differences, alongside increased use of biomarkers, will be required to improve diagnostic accuracy and reduce sex disparities in identifying LBD in the clinical setting.

Clinicopathological concordance for DLB was quite poor with almost a third of participants who were diagnosed with DLB lacking LBP at autopsy, in line with previous studies.^97^ There were no significant differences in the neuropathological burden associated with clinical diagnoses of DLB and PDD but this sub-analysis was likely underpowered statistically. Neocortical LBP was the subtype with the highest percentage of each core clinical features of DLB but there was heterogeneity across clinical symptoms. Furthermore, variability in diagnostic performance across cohorts highlights the need for harmonisation of diagnostic protocols and greater integration of biomarkers to improve detection in clinical populations.^4,36,37,98^

### Strengths and limitations

Strengths of this study include the aggregation of data from nine high-quality community-based autopsy studies to explore the prevalence of LBP in the largest sample to date with neuropathologic workup also differentiated by LBP anatomical distribution. The population- and community-based design of the included cohorts reduces the circularity inherent in many clinicopathological studies allowing less biased assessment of the relationship between pathology and clinical parameters, and avoiding selection of more severe and specific clinical phenotypes. While none of the included cohorts were recruited from dementia- or movement disorder-specific clinical settings, their human population representativeness varied. For example, ROS-MAP specifically recruited religious clergy, while BAS-USP included a younger, more diverse population with a higher proportion of CU individuals at death. The biological and cultural variation in age, geography, cognition and ethnoracial parameters likely contributed to heterogeneity across studies but despite methodological differences, the aggregate *APOE* ε4 prevalence in this study (27%) was close to population-based estimates,^99,100^ suggesting the cohorts were broadly unselected. This study thus was able to discern significant clinical-biological phenomena that would have been impossible to detect using smaller cohorts.

However, several important caveats should be considered. Neuropathological protocols and assessment methods varied across cohorts. One cohort staged LBP using Braak PD criteria, which were approximated to McKeith staging for this analysis and may have led to misclassification in some cases. Cohorts also used different α-synuclein antibodies (Supplementary Table 1); however, prior studies suggest that staging reliability is not dependent on antibody choice,^17^ and all sites used state-of-the-art diagnostic methods. Selection bias related to consent to autopsy is another limitation, with variation in the proportion of the recruited population who were neuropathologically examined across cohorts. Sensitivity analyses performed in some cohorts suggest minimal impact of selection bias but these data were not collected by all cohorts. Another concern related to the current study was that most cohorts reported only summary data, precluding individual-level regression analyses. There was also notable between-study heterogeneity, including differences in cognitive assessments and dementia classification. While differences in study design and geographic location were explored, and no significant differences in LBP prevalence were observed between population-based and community-based cohorts or between U.S. and non-U.S. cohorts, global geographic variation could not be fully assessed due to the predominance of U.S. cohorts (6/9), with only two European and one Brazilian cohort included. Except for the BAS cohort, participants were predominantly White and highly educated, limiting generalisability to more diverse populations and restricting analysis of the impact of ethnicity and social or structural determinants of health. Future studies should aim to investigate LBP prevalence in post-mortem populations with greater ethnic, social, and educational diversity. Additionally, associations between *APOE* ε4 allele positivity and LBP could not be examined due to the absence of participant-level joint distributions. Some sub-analyses were underpowered, and for most cohorts, clinical information beyond cognitive status was unavailable for participants with LBP, with motor symptoms not systematically reported. Finally, post-mortem data represent a single time point and do not capture the dynamic evolution of neuropathology across the lifespan; in vivo biomarkers have yet to be routinely incorporated into large, longitudinal, community-based studies.

## Conclusion

This study establishes robust estimates of LBP prevalence across anatomical subtypes, stratified by cognition, sex, and co-pathologies. As expected, neocortical LBP pathology had the strongest association with dementia with frequent co-morbid ADNC and LATE-NC, each contributing additively to dementia risk. However, there was also marked clinical and neuropathological heterogeneity of LBP, with brainstem-predominant LBP more frequent in men without increased ADNC while amygdala-predominant LBP was more common in women and strongly associated with ADNC. Thus, distinct anatomical distributions of LBP were associated with different demographic and neuropathological signatures. Improving recognition of sex-specific and mixed disease presentations, with wider implementation of biomarkers to support diagnoses, will be critical in reducing underdiagnosis of LBP and advancing precision medicine for clinical trials and for routine clinical management.

## Supplementary Material

Suppl Table 1

Additional supplemental material (1-11)

Supplementary material is available at *Brain* online.

## Figures and Tables

**Figure 1:**
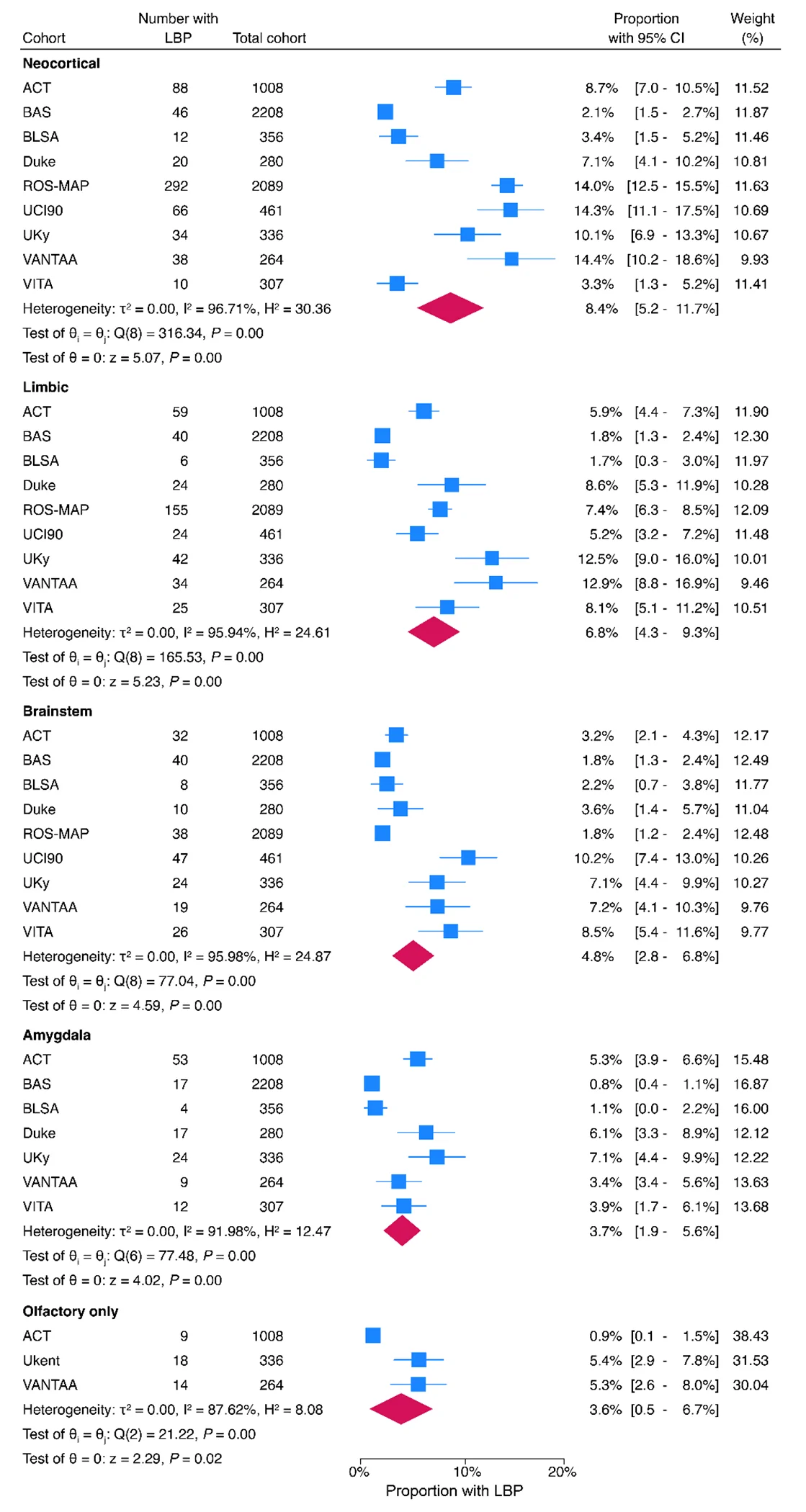
Meta-analysis of proportion of participants with Lewy body pathology for each anatomical distribution. LBP = Lewy body pathology; ACT = the Adult Changes in Thought (ACT) study; BAS-USP = Biobank for Aging Studies from the University of Sao Paulo; BLSA = Baltimore Longitudinal Study of Aging; Duke-UNC ADRC = Duke University/University of North Carolina Alzheimer’s Disease Research Center; ROS-MAP = Rush University Religious Orders Study/Memory and Aging Project; UCI 90+ = University of California, Irvine 90+ Study; UKy-ADRC = University of Kentucky Alzheimer’s Disease Research Center; VITA = Vienna Trans-Danube Aging Study.

**Figure 2:**
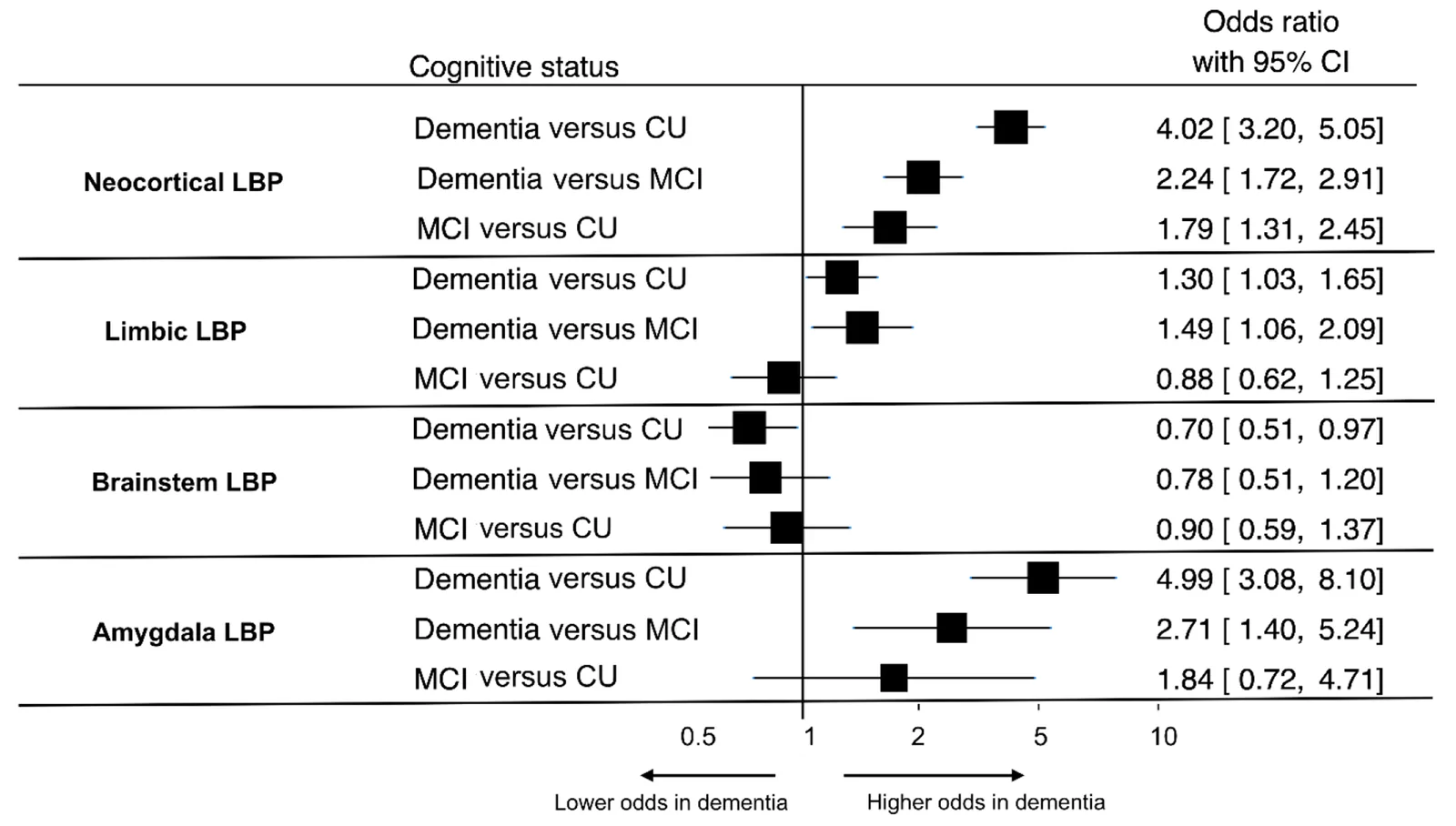
Odds of each subtype of LBP by cognitive status. LBP = Lewy body pathology; MCI = mild cognitive impairment; CU = cognitively unimpaired.

**Figure 3:**
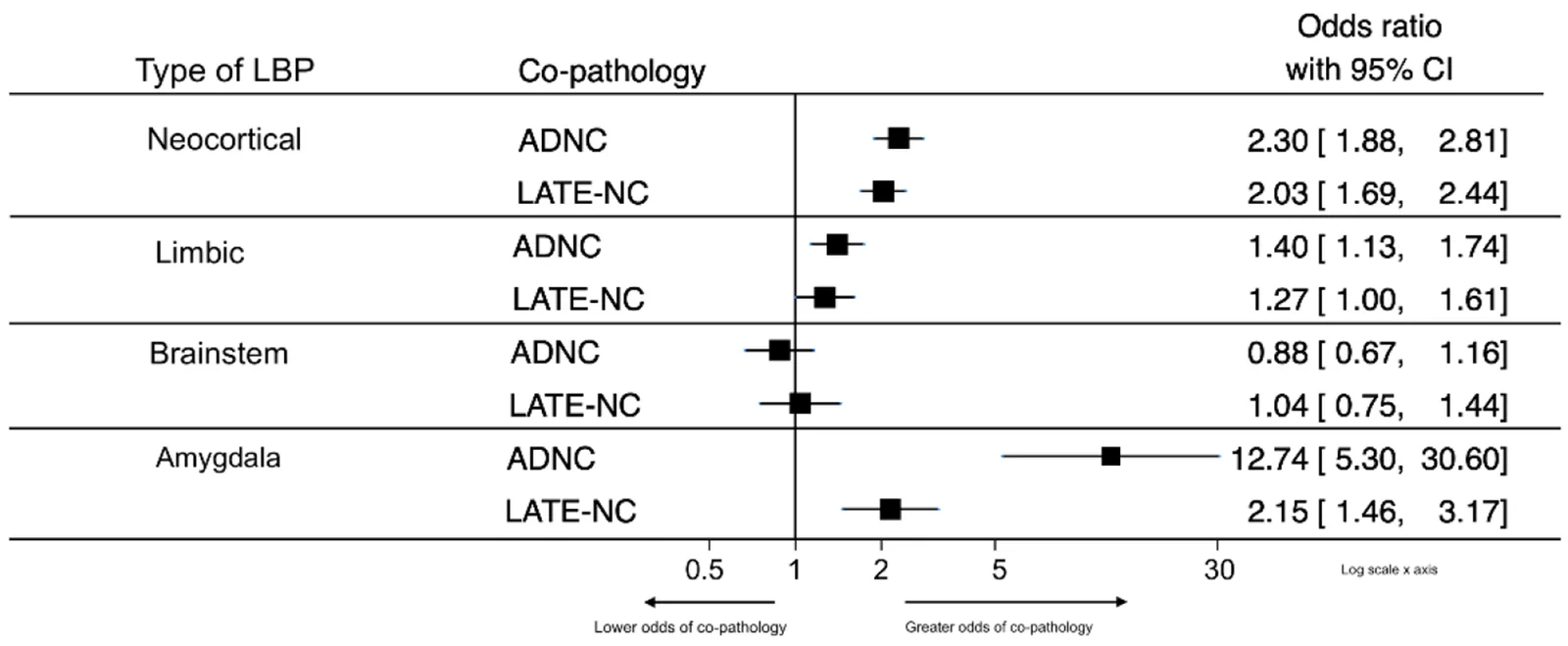
Odds of ADNC and LATE-NC co-pathology in each subtype of LBP. LBP = Lewy body pathology; ADNC = Alzheimer’s disease neuropathological change; LATE-NC = limbic-predominant age-related TDP-43 encephalopathy neuropathological change.

**Table 1:** Summary demographic and neuropathological characteristics of each cohort

Cohort characteristics	ACT	BAS-USP	BLSA	Duke-UNC ADRC	ROS-MAP	UCI 90+	UKy-ADRC	Vantaa 85+	VITA	Total sample
Country	USA	Brazil	USA	USA	USA	USA	USA	Finland	Austria	
Sample size	1008	2208	356	280	2089	461	336	264	307	7309
Average age at death, yrs (SD)	89	72.9 (13.9)	87	84 (10.8)	89.5 (6.8)	97.7 (3.6)	83.9 (9.6)	92.6 (3.6)	83.2 (3.3)	84.2
% women, n	58.6% (591)	49.3%(1091)	37.9% (135)	59.6% (167)	68.8% (1437)	66.6% (307)	56% (188)	82.6% (218)	58.6% (180)	59% (4314)
# with known APOE status	1008	1354	-	271	1980	461	317	264	-	5655
APOE ε4 allele present, %, n	27.5% (277)	29.0% (393)	-	30.3% (82)	25.5% (505)	18.4% (85)	40.4% (128)	32.2% (85)	-	27.5% (1556)
Last cognitive status before death, %, n										
Cognitively unimpaired	50.4% (508)	67.2% (1476)	-	36.6% (101)	34% (714)	28.9% (133)	26.9% (87)	34.8% (92)	-	47% (3111)
Mild cognitive impairment	2.1% (21)	9.5% (209)	-	18.8% (52)	23% (477)	28.4% (131)	15.8% (51)	-	-	14.8% (941)
Clinical dementia	45.7% (461)	23.3% (511)	-	44.6% (123)	43% (897)	42.7% (197)	57.3% (185)	65.2% (172)	-	38.5% (2546)
LBP, %, n										
Neocortical	8.7% (88)	2.1% (46)	3.4% (12)	7.1% (20)	14% (292)	14.3% (66)	10% (34)	14.4% (38)	3.3% (10)	8.3% (606)
Limbic-predominant	5.9% (59)	1.8% (40)	1.7% (6)	8.6% (24)	7.4% (155)	5.2% (24)	12.5% (42)	12.9% (34)	8.1% (25)	5.6% (409)
Brainstem-predominant	3.2% (32)	1.8% (40)	2.2% (8)	3.6% (10)	1.8% (38)	10.2% (47)	7.1% (24)	7.2% (19)	8.5% (26)	3.3% (244)
Amygdala-predominant	5.3% (53)	0.8% (17)	1.1% (4)	6.1% (17)	-	-	7.1% (24)	3.4% (9)	3.9% (12)	2.9% (136)
Olfactory only	0.9% (9)		-	-	-	-	5.4% (18)		5.3% (14)	2.5% (41)
Thal phase > 3, %, n	48.3% (487)	16.5% (325)	34% (121)	44% (123)	66% (1379)	53% (242)	57% (149)	70.5% (186)	12.4% (38)	43.6% (3050)
CERAD Moderate %, n	26.8% (270)	13.7% (303)	36.5% (130)	32.9% (92)	37% (776)	12% (57)	12% (41)	53.8% (142)	22.1% (68)	24.6% (1795)
CERAD Frequent %, n	28.9% (291)	10.5% (231)	19.1% (68)	8.9% (25)	30% (636)	52% (239)	43% (143)	12.5% (33)	14.3% (44)	22.7% (1655)
Braak NFT V-VI %, n	38.8% (391)	12.4% (272)	18.2% (65)	20.0% (56)	26% (543)	38% (173)	50% (161)	35.6% (94)	11.4% (35)	24.6% (1790)
LATE-NC stage >1 %, n	27.6% (278)	4.9% (91)	33.1% (118)	11.1% (31)	35% (685)	30% (140)	31% (93)	41.7% (110)	10.4% (32)	23.2% (1578)

ACT = the Adult Changes in Thought (ACT) study; BAS-USP = Biobank for Aging Studies from the University of Sao Paulo; BLSA = Baltimore Longitudinal Study of Aging; Duke-UNC ADRC = Duke University/University of North Carolina Alzheimer’s Disease Research Center; ROS-MAP = Rush University Religious Orders Study/Memory and Aging Project; UCI 90+ = University of California, Irvine 90+ Study; UKy-ADRC = University of Kentucky Alzheimer’s Disease Research Center; VITA = Vienna Trans-Danube Aging Study. LBP = Lewy body pathology; CERAD = Consortium to Establish a Registry for Alzheimer’s Disease; NFT = neurofibrillary tangle; LATE-NC = Limbic-predominant age-related TDP-43 encephalopathy neuropathological change.

**Table 2:** Participants with each subtype of LBP in stratified by final cognitive status

LBP subtype	Dementia		Mild cognitive impairment		Cognitively unimpaired		p value
	Total number across cohorts (n=2546)	Meta analysis proportion [95% CI]	Total number across cohorts (n=941)	Meta analysis proportion [95% CI]	Total number across cohorts (n=3111)	Meta analysis proportion [95% CI]	
Neocortical	399	15% [11–19%]	78	8% [4–11%]	105	4% [2–6%]	**<0.001**
Limbic	193	8% [5–11%]	47	4% [2–7%]	135	6% [4–9%]	**<0.001**
rainstem	76	4% [1–6%]	34	5% [1–9%]	100	5% [3–7%]	0.638
Amygdala^a^	92	7% [4–10%]	6	2% [0–4%]	22	1% [0–2%]	**<0.001**
Olfactory only^b^	26 (3.2%)	–	2 (2.8%)	–	13 (1.9%)	–	0.294

The total number of participants with each subtype of LBP in stratified by final cognitive status. Proportions were calculated from meta-analysis stratified by cognitive status.The difference in proportions across cognitive groups was evaluated using χ^2^ tests, significant results highlighted in bold if p<0.05.

LBP = Lewy body pathology.

a Participants with assessment for amygdala LBP: dementia (n=1452), mild cognitive impairment (n=333), cognitively unimpaired (n=2264).

b Participants with assessment for olfactory LBP: dementia (n=818), mild cognitive impairment (n=72), cognitively unimpaired (n =687). Meta-analysis not conducted for olfactory only LBP because only 2 cohorts with cognitive data reported olfactory only LBP.

**Table 3:** Proportion with each staging of co-pathology across participants separated by anatomical distribution of LBP

Staging of co-pathology	All participants (n=7309)	Anatomical distribution of LBP					p value
		Neocortical (n=606)	Limbic-predominant (n=409)	Brainstem-predominant (n=244)	Amygdala-predominant (n=137)	Olfactory only (n=41)	
**Braak NFT staging** ^a^							
0-II	75.4% (5497)	11.3% (68)	17.6% (72)	28.5% (69)	8.9% (12)	10.0% (4)	**<0.001**
III-IV		46.4% (280)	48.7% (199)	55.4% (134)	17.8% (24)	25.0% (10)	**<0.001**
V-VI	24.6% (1758)	42.2% (255)	33.7% (138)	16.1% (39)	73.3% (99)	65.0% (26)	**<0.001**
**CERAD neuritic amyloid plaque stages**							
None	52.8% (3854)	18.2% (110)	27.4% (112)	38.5% (94)	5.1% (7)	17.1% (7)	**<0.001**
Sparse		10.6% (64)	14.9% (61)	16.8% (41)	10.9% (15)	9.8% (4)	0.073
Moderate	26.3% (1795)	32.3% (196)	27.1% (111)	23.8% (58)	38.0% (52)	26.8% (11)	**0.017**
Frequent	22.7% (1655)	38.8% (236)	30.6% (125)	20.9% (51)	46.0% (63)	46.3% (19)	**<0.001**
**Thal Aβphase** ^b^							
0	23.8% (1666)	11.1% (66)	13.2% (53)	21.7% (52)	1.5% (2)	7.9% (3)	**<0.001**
1-2	32.5% (2274)	11.9% (71)	23.4% (94)	21.7% (52)	8.1% (11)	10.5% (4)	**<0.001**
3		19.9% (119)	17.2% (69)	22.5% (54)	9.6% (13)	13.2% (5)	**0.019**
4-5	43.6% (3050)	57.1% (341)	46.3% (186)	34.2% (82)	80.9% (110)	68.4% (26)	**<0.001**
**ADNC** ^c^							
Mild or absent	51.2% (3450)	25.7% (153)	38.9% (157)	52.3% (125)	11.3% (6)	-	**<0.001**
Moderate or severe	48.8% (3289)	74.3% (442)	61.1% (247)	47.7% (114)	88.7% (47)	-	**<0.001**
**LATE-NC (TDP-43) stages** ^d^							
Stage 0	66.7% (5226)	40.4% (241)	52.9% (211)	63.7% (151)	43.1% (59)	26.3% (10)	**<0.001**
Stage 1		17.3% (103)	17.8% (71)	13.9% (33)	23.4% (32)	23.7% (9)	0.17
Stage 2-3	23.3% (1578)	42.3% (252)	29.3% (117)	22.4% (53)	33.6% (46)	50.0% (19)	**<0.001**

Differences in proportions across groups were assessed in X^2^ tests with significant in bold at the 5% level.

LBP = Lewy body pathology; NFT = neurofibrillary tangle; CERAD = Consortium to Establish a Registry for Alzheimer’s Disease; ADNC = Alzheimer’s disease neuropathological change; LATE-NC = Limbic-predominant age-related TDP-43 encephalopathy neuropathological change

a Number missing across each subgroup: neocortical n=3; limbic n=2; brainstem n=2; amygdala n=2 olfactory n=1.

b Number missing across each subgroup: neocortical n=9; limbic n=7; brainstem n=2; amygdala n=1; olfactory n=3.

c Number missing in each subgroup: neocortical n=11, limbic n=5, brainstem n=5, amygdala n=84.

d Number missing across each subgroup: neocortical n=10; limbic n=10; brainstem n=7; amygdala n=1; olfactory n=3.

**Table 4:** Co-pathology stratified by cognitive status across each anatomical subtype of LBP

		ADNC present (n=2020)	LATE-NC present	p ADNC	p LATE
Neocortical LBP (n=368)^a^	Dementia (249)	82.9% (204)	47.4% (118)	<0.001	0.003
	MCI (56)	56.4% (31)	33.9% (19)		
	CU (63)	48.4% (30)	25.4% (16)		
Limbic LBP (n=234)^b^	Dementia (n=124)	81.8% (99)	45.2% (56)	<0.001	<0.001
	MCI (n=40)	56.4% (22)	25.0% (10)		
	CU (n=70)	37.1% (26)	11.4% (8)		
Brainstem LBP (n=107)^c^	Dementia (n=31)	51.6% (16)	38.7% (12)	0.020	0.001
	MCI (n=19)	41.2% (7)	26.3% (5)		
	CU (n=57)	22.8% (13)	5.5% (3)		
Amygdala LBP (n=51) ^d^	Dementia (n=40)	100% (39)	52.5% (21)	<0.001	0.033
	MCI (n=4)	75% (3)	0		
	CU (n=7)	33% (2)	14.3% (1)		
No LBP (n=3621)^e^	Dementia (n=1092)	68.9% (752)	36.2% (366)	<0.001	<0.001
	MCI (n=620)	49.5% (307)	24.1% (146)		
	CU (n=1909)	23.8% (454)	7.2% (126)		

Comparisons across groups conducted with X^2^ tests.

ADNC = Alzheimer’s disease neuropathological change; LATE-NC = Limbic-predominant age-related TDP-43 encephalopathy neuropathological change; LBP = Lewy body pathology, MCI = mild cognitive impairment; CU = cognitively unimpaired; Reduced numbers reported for some subgroups across the cohorts

a Neocortical LBP, ADNC reported in: dementia n=246, MCI n=55, CU n=62.

b Limbic LBP, ADNC reported in: dementia n=121, MCI n=39.

c Brainstem LBP, ADNC reported in MCI n=17, LATE-NC reported in CU n=55.

d Amygdala LBP reported in ADNC dementia n=39, CU n=6.

e No LBP reported in LATE-NC dementia n=1012, MCI n=605, CU n=1758.

**Table 5:** Prevalence of each subtype of LBP and other neurodegenerative pathologies stratified across men and women

Pathologic features		Men		Women		p value
		Total number across cohorts (n=2995)	Meta analysis proportion [95% CI]	Total number across cohorts (n=4314)	Meta analysis proportion [95% CI]	
LBP subtype	Neocortical	230 (7.7%)	9% [5–13%]	376 (8.7%)	8% [5–11%]	0.11
	Limbic	162 (5.4%)	7% [4–10%]	247 (5.7%)	6% [4–9%]	0.56
	Brainstem	119 (4.0%)	5% [3–6%]	125 (2.9%)	4% [2–6%]	**0.012**
	Amygdala^a^	45 (2.1%)	3% [1–5%]	92 (3.6%)	4% [2–6%]	**0.002**

Other neurodegenerative pathologies		Total across cohorts (%)		Total across cohorts (%)		
Thal Aβphases 4 or 5^b^		913 (36.5%)		1978 (51.7%)		<0.001
Braak NFT stages V or VI^c^		504 (19.1%)		1186 (29.7%)		<0.001
CERAD neuritic plaque stages moderate or frequent^d^		1058 (40%)		2210 (55.3%)		**0.003**
LATE-NC stages 2 or 3^e^		424 (17.6%)		1004 (26.9%)		<0.001

Difference in proportions across sex were evaluated in χ^2^ tests, significant results highlighted in bold if p<0.05.

CI = confidence interval; LBP = Lewy body pathology; NFT = neurofibrillary tangle; CERAD = Consortium to Establish a Registry for Alzheimer’s Disease; LATE-NC = Limbic-predominant age-related TDP-43 encephalopathy neuropathological change

Number of men and women participants each neurodegenerative pathology was reported in:

a Amygdala-predominant LBP: men, n=2189, women n=2570.

b Thal phase men n= 2504, women n=3823.

c Braak NFT staging men n=2637, women n=3987.

d CERAD staging, men n=2645, women n=3998 .

e LATE-NC staging, men n=2413, women=3728.

## Data Availability

The data in this study is collated from nine international cohorts. The data that support the findings of this study are available from the corresponding author, upon reasonable request.

## References

[1] Vann Jones SA, O’Brien JT. The prevalence and incidence of dementia with Lewy bodies: a systematic review of population and clinical studies. Psychol Med. Mar 2014;44(4):673–83. doi:10.1017/S0033291713000494 23521899

[2] Aarsland D, Rongve A, Nore SP, Frequency and case identification of dementia with Lewy bodies using the revised consensus criteria. Dement Geriatr Cogn Disord. 2008;26(5):445–52. doi:10.1159/000165917 18974647

[3] Surendranathan A, Kane JPM, Bentley A, Clinical diagnosis of Lewy body dementia. BJPsych Open. Jun 16 2020;6(4):e61. doi:10.1192/bjo.2020.44 PMC7345593 32539875

[4] Kane JPM, Surendranathan A, Bentley A, Clinical prevalence of Lewy body dementia. Alzheimers Res Ther. Feb 15 2018;10(1):19. doi:10.1186/s13195-018-0350-6 PMC5815202 29448953

[5] Parkkinen L, Pirttila T, Alafuzoff I. Applicability of current staging/categorization of alpha-synuclein pathology and their clinical relevance. Acta Neuropathol. Apr 2008;115(4):399–407. doi:10.1007/s00401-008-0346-6 PMC2270355 18297293

[6] Gibb WR, Lees AJ. The relevance of the Lewy body to the pathogenesis of idiopathic Parkinson’s disease. J Neurol Neurosurg Psychiatry. Jun 1988;51(6):745–52. doi:10.1136/jnnp.51.6.745 PMC1033142 2841426

[7] Forrest SL, Kovacs GG. Current Concepts of Mixed Pathologies in Neurodegenerative Diseases. Can J Neurol Sci. May 2023;50(3):329–345. doi:10.1017/cjn.2022.34 35356856

[8] Zaccai J, Brayne C, McKeith I, Matthews F, Ince PG, Mrc Cognitive Function ANS. Patterns and stages of alpha-synucleinopathy: Relevance in a population-based cohort. Neurology. Mar 25 2008;70(13):1042–8. doi:10.1212/01.wnl.0000306697.48738.b6 18362284

[9] Wakisaka Y, Furuta A, Tanizaki Y, Kiyohara Y, Iida M, Iwaki T. Age-associated prevalence and risk factors of Lewy body pathology in a general population: the Hisayama study. Acta Neuropathol. Oct 2003;106(4):374–82. doi:10.1007/s00401-003-0750-x 12904992

[10] Parkkinen L, Soininen H, Laakso M, Alafuzoff I. Alpha-synuclein pathology is highly dependent on the case selection. Neuropathol Appl Neurobiol. Aug 2001;27(4):314–25. doi:10.1046/j.0305-1846.2001.00342.x 11532162

[11] Neuropathology Group. Medical Research Council Cognitive F, Aging S. Pathological correlates of late-onset dementia in a multicentre, community-based population in England and Wales. Neuropathology Group of the Medical Research Council Cognitive Function and Ageing Study (MRC CFAS). Lancet. Jan 20 2001;357(9251):169–75. doi:10.1016/s0140-6736(00)03589-3 11213093

[12] Kovacs GG, Milenkovic I, Wohrer A, Non-Alzheimer neurodegenerative pathologies and their combinations are more frequent than commonly believed in the elderly brain: a community-based autopsy series. Acta Neuropathol. Sep 2013;126(3):365–84. doi:10.1007/s00401-013-1157-y 23900711

[13] Schneider JA, Arvanitakis Z, Bang W, Bennett DA. Mixed brain pathologies account for most dementia cases in community-dwelling older persons. Neurology. Dec 11 2007;69(24):2197–204. doi:10.1212/01.wnl.0000271090.28148.24 17568013

[14] Wang X, Zhu K, Wu W, Prevalence of mixed neuropathologies in age-related neurodegenerative diseases: A community-based autopsy study in China. Alzheimers Dement. Jan 2025;21(1):e14369. doi:10.1002/alz.14369 PMC11782840 39582417

[15] Braak H, Del Tredici K, Rub U, de Vos RA, Jansen Steur EN, Braak E. Staging of brain pathology related to sporadic Parkinson’s disease. Neurobiol Aging. Mar-Apr 2003;24(2):197–211. doi:10.1016/s0197-4580(02)00065-9 12498954

[16] McKeith IG, Dickson DW, Lowe J, Diagnosis and management of dementia with Lewy bodies: third report of the DLB Consortium. Neurology. Dec 27 2005;65(12):1863–72. doi:10.1212/01.wnl.0000187889.17253.b1 16237129

[17] Attems J, Toledo JB, Walker L, Neuropathological consensus criteria for the evaluation of Lewy pathology in post-mortem brains: a multi-centre study. Acta Neuropathol. Feb 2021;141(2):159–172. doi:10.1007/s00401-020-02255-2 PMC7847437 33399945

[18] Boyle PA, Yu L, Wilson RS, Schneider JA, Bennett DA. Relation of neuropathology with cognitive decline among older persons without dementia. Front Aging Neurosci. 2013;5:50. doi:10.3389/fnagi.2013.00050 PMC3766823 24058343

[19] Coughlin DG, Hurtig HI, Irwin DJ. Pathological Influences on Clinical Heterogeneity in Lewy Body Diseases. Mov Disord. Jan 2020;35(1):5–19. doi:10.1002/mds.27867 PMC7233798 31660655

[20] Jellinger KA, Attems J. Prevalence and pathology of dementia with Lewy bodies in the oldest old: a comparison with other dementing disorders. Dement Geriatr Cogn Disord. 2011;31(4):309–16. doi:10.1159/000327360 21502762

[21] Almeida FC, Santos A, Jesus T, Amygdala-predominant Lewy bodies associate with cognitive decline and amygdala atrophy in Alzheimer’s disease neuropathological change. J Neurol. Sep 12 2025;272(9):629. doi:10.1007/s00415-025-13360-1 PMC13167139 40940550

[22] Rahimi J, Kovacs GG. Prevalence of mixed pathologies in the aging brain. Alzheimers Res Ther. 2014;6(9):82. doi:10.1186/s13195-014-0082-1 PMC4239398 25419243

[23] Schneider JA, Aggarwal NT, Barnes L, Boyle P, Bennett DA. The neuropathology of older persons with and without dementia from community versus clinic cohorts. J Alzheimers Dis. 2009;18(3):691–701. doi:10.3233/JAD-2009-1227 PMC2853869 19749406

[24] Zaccai J, Ince P, Brayne C. Population-based neuropathological studies of dementia: design, methods and areas of investigation--a systematic review. BMC Neurol. Jan 9 2006;6:2. doi:10.1186/1471-2377-6-2 PMC1397861 16401346

[25] Spires-Jones TL, Attems J, Thal DR. Interactions of pathological proteins in neurodegenerative diseases. Acta Neuropathol. Aug 2017;134(2):187–205. doi:10.1007/s00401-017-1709-7 PMC5508034 28401333

[26] Ubhi K, Peng K, Lessig S, Neuropathology of dementia with Lewy bodies in advanced age: a comparison with Alzheimer disease. Neurosci Lett. Nov 26 2010;485(3):222–7. doi:10.1016/j.neulet.2010.09.016 PMC2987680 20849919

[27] Chiu SY, Wyman-Chick KA, Ferman TJ, Sex differences in dementia with Lewy bodies: Focused review of available evidence and future directions. Parkinsonism Relat Disord. Feb 2023;107:105285. doi:10.1016/j.parkreldis.2023.105285 PMC10024862 36682958

[28] Savica R, Grossardt BR, Bower JH, Boeve BF, Ahlskog JE, Rocca WA. Incidence of dementia with Lewy bodies and Parkinson disease dementia. JAMA Neurol. Nov 2013;70(11):1396–402. doi:10.1001/jamaneurol.2013.3579 PMC4181848 24042491

[29] Bayram E, Coughlin DG, Banks SJ, Litvan I. Sex differences for phenotype in pathologically defined dementia with Lewy bodies. J Neurol Neurosurg Psychiatry. Jul 2021;92(7):745–750. doi:10.1136/jnnp-2020-325668 PMC8530264 33563809

[30] Cerri S, Mus L, Blandini F. Parkinson’s Disease in Women and Men: What’s the Difference? J Parkinsons Dis. 2019;9(3):501–515. doi:10.3233/JPD-191683 PMC6700650 31282427

[31] Fereshtehnejad SM, Religa D, Westman E, Aarsland D, Lokk J, Eriksdotter M. Demography, diagnostics, and medication in dementia with Lewy bodies and Parkinson’s disease with dementia: data from the Swedish Dementia Quality Registry (SveDem). Neuropsychiatr Dis Treat. 2013;9:927–35. doi:10.2147/NDT.S45840 PMC3700781 23847419

[32] Nelson PT, Schmitt FA, Jicha GA, Association between male gender and cortical Lewy body pathology in large autopsy series. J Neurol. Nov 2010;257(11):1875–81. doi:10.1007/s00415-010-5630-4 PMC3040648 20563821

[33] Toledo JB, Abdelnour C, Weil RS, Dementia with Lewy bodies: Impact of co-pathologies and implications for clinical trial design. Alzheimers Dement. Jan 2023;19(1):318–332. doi:10.1002/alz.12814 PMC9881193 36239924

[34] Irwin DJ, Grossman M, Weintraub D, Neuropathological and genetic correlates of survival and dementia onset in synucleinopathies: a retrospective analysis. Lancet Neurol. Jan 2017;16(1):55–65. doi:10.1016/S1474-4422(16)30291-5 PMC5181646 27979356

[35] Robinson JL, Xie SX, Baer DR, Pathological combinations in neurodegenerative disease are heterogeneous and disease-associated. Brain. Mar 2 2023;doi:10.1093/brain/awad059 PMC10232273 36864661

[36] McKeith IG, Boeve BF, Dickson DW, Diagnosis and management of dementia with Lewy bodies: Fourth consensus report of the DLB Consortium. Neurology. Jul 4 2017;89(1):88–100. doi:10.1212/WNL.0000000000004058 PMC5496518 28592453

[37] McAleese KE, Colloby SJ, Thomas AJ, Concomitant neurodegenerative pathologies contribute to the transition from mild cognitive impairment to dementia. Alzheimers Dement. Jul 2021;17(7):1121–1133. doi:10.1002/alz.12291 33663011

[38] McAleese KE, Walker L, Erskine D, Thomas AJ, McKeith IG, Attems J. TDP-43 pathology in Alzheimer’s disease, dementia with Lewy bodies and ageing. Brain Pathol. Jul 2017;27(4):472–479. doi:10.1111/bpa.12424 PMC8029292 27495267

[39] Robinson JL, Lee EB, Xie SX, Neurodegenerative disease concomitant proteinopathies are prevalent, age-related and APOE4-associated. Brain. Jul 1 2018;141(7):2181–2193. doi:10.1093/brain/awy146 PMC6022546 29878075

[40] Bayram E, Shan G, Cummings JL. Associations between Comorbid TDP-43, Lewy Body Pathology, and Neuropsychiatric Symptoms in Alzheimer’s Disease. J Alzheimers Dis. 2019;69(4):953–961. doi:10.3233/JAD-181285 PMC6597983 31127776

[41] Arai T, Mackenzie IR, Hasegawa M, Phosphorylated TDP-43 in Alzheimer’s disease and dementia with Lewy bodies. Acta Neuropathol. Feb 2009;117(2):125–36. doi:10.1007/s00401-008-0480-1 19139911

[42] Nakashima-Yasuda H, Uryu K, Robinson J, Co-morbidity of TDP-43 proteinopathy in Lewy body related diseases. Acta Neuropathol. Sep 2007;114(3):221–9. doi:10.1007/s00401-007-0261-2 17653732

[43] Boyle PA, Yang J, Yu L, Varied effects of age-related neuropathologies on the trajectory of late life cognitive decline. Brain. Mar 1 2017;140(3):804–812. doi:10.1093/brain/aww341 PMC5837473 28082297

[44] Ryman SG, Yutsis M, Tian L, Cognition at Each Stage of Lewy Body Disease with Co-occurring Alzheimer’s Disease Pathology. J Alzheimers Dis. 2021;80(3):1243–1256. doi:10.3233/JAD-201187 PMC8150665 33646154

[45] Gibson LL, Grinberg LT, Ffytche D, Neuropathological correlates of neuropsychiatric symptoms in dementia. Alzheimers Dement. Sep 23 2022;doi:10.1002/alz.12765 PMC10033459 36150075

[46] Coughlin D, Xie SX, Liang M, Cognitive and Pathological Influences of Tau Pathology in Lewy Body Disorders. Ann Neurol. Feb 2019;85(2):259–271. doi:10.1002/ana.25392 PMC6375484 30549331

[47] Peavy GM, Edland SD, Toole BM, Hansen LA, Galasko DR, Mayo AM. Phenotypic differences based on staging of Alzheimer’s neuropathology in autopsy-confirmed dementia with Lewy bodies. Parkinsonism Relat Disord. Oct 2016;31:72–78. doi:10.1016/j.parkreldis.2016.07.008 PMC5794000 27475955

[48] Chin KS, Yassi N, Churilov L, Masters CL, Watson R. Prevalence and clinical associations of tau in Lewy body dementias: A systematic review and meta-analysis. Parkinsonism Relat Disord. Nov 2020;80:184–193. doi:10.1016/j.parkreldis.2020.09.030 33260030

[49] Donaghy PC, Firbank MJ, Thomas AJ, Amyloid Imaging and Longitudinal Clinical Progression in Dementia With Lewy Bodies. Am J Geriatr Psychiatry. May 2020;28(5):573–577. doi:10.1016/j.jagp.2019.12.009 31953009

[50] Sarro L, Senjem ML, Lundt ES, Amyloid-beta deposition and regional grey matter atrophy rates in dementia with Lewy bodies. Brain. Oct 2016;139(Pt 10):2740–2750. doi:10.1093/brain/aww193 PMC5035818 27452602

[51] Uchikado H, Lin WL, DeLucia MW, Dickson DW. Alzheimer disease with amygdala Lewy bodies: a distinct form of alpha-synucleinopathy. J Neuropathol Exp Neurol. Jul 2006;65(7):685–97. doi:10.1097/01.jnen.0000225908.90052.07 PMC5706655 16825955

[52] Borghammer P, Horsager J, Andersen K, Neuropathological evidence of body-first vs. brain-first Lewy body disease. Neurobiol Dis. Dec 2021;161:105557. doi:10.1016/j.nbd.2021.105557 34763110

[53] Raunio A, Kaivola K, Tuimala J, Lewy-related pathology exhibits two anatomically and genetically distinct progression patterns: a population-based study of Finns aged 85. Acta Neuropathol. Nov 2019;138(5):771–782. doi:10.1007/s00401-019-02071-3 PMC6800868 31494694

[54] Kukull WA, Higdon R, Bowen JD, Dementia and Alzheimer disease incidence: a prospective cohort study. Arch Neurol. Nov 2002;59(11):1737–46. doi:noc20207 [pii] 10.1001/archneur.59.11.1737 12433261

[55] Mikolaenko I, Pletnikova O, Kawas CH, Alpha-synuclein lesions in normal aging, Parkinson disease, and Alzheimer disease: evidence from the Baltimore Longitudinal Study of Aging (BLSA). J Neuropathol Exp Neurol. Feb 2005;64(2):156–62. doi:10.1093/jnen/64.2.156 15751230

[56] O’Brien RJ, Resnick SM, Zonderman AB, Neuropathologic studies of the Baltimore Longitudinal Study of Aging (BLSA). J Alzheimers Dis. 2009;18(3):665–75. doi:10.3233/JAD-2009-1179 PMC2978421 19661626

[57] Suemoto CK, Leite REP, Ferretti-Rebustini REL, Neuropathological lesions in the very old: results from a large Brazilian autopsy study. Brain Pathol. Nov 2019;29(6):771–781. doi:10.1111/bpa.12719 PMC6742578 30861605

[58] Suemoto CK, Ferretti-Rebustini RE, Rodriguez RD, Neuropathological diagnoses and clinical correlates in older adults in Brazil: A cross-sectional study. PLoS Med. Mar 2017;14(3):e1002267. doi:10.1371/journal.pmed.1002267 PMC5369698 28350821

[59] Grinberg LT, Ferretti RE, Farfel JM, Brain bank of the Brazilian aging brain study group - a milestone reached and more than 1,600 collected brains. Cell Tissue Bank. 2007;8(2):151–62. doi:10.1007/s10561-006-9022-z 17075689

[60] Wang SJ, Guo Y, Ervin JF, Lusk JB, Luo S. Neuropathological associations of limbic-predominant age-related TDP-43 encephalopathy neuropathological change (LATE-NC) differ between the oldest-old and younger-old. Acta Neuropathol. Jul 2022;144(1):45–57. doi:10.1007/s00401-022-02432-5 PMC9997084 35551470

[61] Bennett DA, Schneider JA, Arvanitakis Z, Wilson RS. Overview and findings from the religious orders study. Curr Alzheimer Res. Jul 2012;9(6):628–45. doi:10.2174/156720512801322573 PMC3409291 22471860

[62] Bennett DA, Buchman AS, Boyle PA, Barnes LL, Wilson RS, Schneider JA. Religious Orders Study and Rush Memory and Aging Project. J Alzheimers Dis. 2018;64(s1):S161–S189. doi:10.3233/JAD-179939 PMC6380522 29865057

[63] Corrada MM, Berlau DJ, Kawas CH. A population-based clinicopathological study in the oldest-old: the 90+ study. Curr Alzheimer Res. Jul 2012;9(6):709–17. 10.2174/156720512801322537 PMC3409303 22471863

[64] Melikyan ZA, Greenia DE, Corrada MM, Hester MM, Kawas CH, Grill JD. Recruiting the Oldest-old for Clinical Research. Alzheimer Dis Assoc Disord. Apr-Jun 2019;33(2):160–162. doi:10.1097/WAD.0000000000000260 PMC6289893 29734262

[65] Schmitt FA, Nelson PT, Abner E, University of Kentucky Sanders-Brown Healthy Brain Aging Volunteers: Donor Characteristics, Procedures, and Neuropathology. Curr Alzheimer Res. Apr 2 2012;9(6):724–33. 10.2174/156720512801322591 PMC3409295 22471862

[66] Schmitt FA, Abner EL, Fardo DW, Celebrating 40 years of the University of Kentucky Alzheimer’s Disease Research Center. Alzheimers Dement. May 2025;21(5):e70181. doi:10.1002/alz.70181 PMC12076195 40365904

[67] Kero M, Raunio A, Polvikoski T, Tienari PJ, Paetau A, Myllykangas L. Hippocampal Sclerosis in the Oldest Old: A Finnish Population-Based Study. J Alzheimers Dis. 2018;63(1):263–272. doi:10.3233/JAD-171068 PMC5900558 29614661

[68] Forrest SL, Kovacs GG. Protein Pathologies in Oligodendroglia in Neurodegenerative Diseases. Adv Neurobiol. 2025;43:407–432. doi:10.1007/978-3-031-87919-7_14 40500505

[69] Fischer P, Jungwirth S, Krampla W, Vienna Transdanube Aging “VITA”: study design, recruitment strategies and level of participation. J Neural Transm Suppl. 2002;(62):105–16. doi:10.1007/978-3-7091-6139-5_11 12456056

[70] Nelson PT, Brayne C, Flanagan ME, Frequency of LATE neuropathologic change across the spectrum of Alzheimer’s disease neuropathology: combined data from 13 community-based or population-based autopsy cohorts. Acta Neuropathol. Jul 2022;144(1):27–44. doi:10.1007/s00401-022-02444-1 PMC9552938 35697880

[71] McKeith IG, Galasko D, Kosaka K, Consensus guidelines for the clinical and pathologic diagnosis of dementia with Lewy bodies (DLB): report of the consortium on DLB international workshop. Neurology. Nov 1996;47(5):1113–24. doi:10.1212/wnl.47.5.1113 8909416

[72] Schneider JA, Arvanitakis Z, Yu L, Boyle PA, Leurgans SE, Bennett DA. Cognitive impairment, decline and fluctuations in older community-dwelling subjects with Lewy bodies. Brain. Oct 2012;135(Pt 10):3005–14. doi:10.1093/brain/aws234 PMC3470712 23065790

[73] Braak H, Braak E. Neuropathological stageing of Alzheimer-related changes. Acta Neuropathol. 1991;82(4):239–59. doi:10.1007/BF00308809 1759558

[74] Mirra SS, Heyman A, McKeel D, The Consortium to Establish a Registry for Alzheimer’s Disease (CERAD). Part II. Standardization of the neuropathologic assessment of Alzheimer’s disease. Neurology. Apr 1991;41(4):479–86. doi:10.1212/wnl.41.4.479 2011243

[75] Thal DR, Capetillo-Zarate E, Del Tredici K, Braak H. The development of amyloid beta protein deposits in the aged brain. Sci Aging Knowledge Environ. Mar 8 2006;2006(6):re1. doi:10.1126/sageke.2006.6.re1 16525193

[76] Montine TJ, Phelps CH, Beach TG, National Institute on Aging-Alzheimer’s Association guidelines for the neuropathologic assessment of Alzheimer’s disease: a practical approach. Acta Neuropathol. Jan 2012;123(1):1–11. doi:10.1007/s00401-011-0910-3 PMC3268003 22101365

[77] Nelson PT, Dickson DW, Trojanowski JQ, Limbic-predominant age-related TDP-43 encephalopathy (LATE): consensus working group report. Brain. Jun 1 2019;142(6):1503–1527. doi:10.1093/brain/awz099 PMC6536849 31039256

[78] Ferretti REL, Damin AE, Brucki SMD, Post-Mortem diagnosis of dementia by informant interview. Dement Neuropsychol. Apr-Jun 2010;4(2):138–144. doi:10.1590/S1980-57642010DN40200011 PMC5619173 29213677

[79] Nunes PV, Schwarzer MC, Leite REP, Neuropsychiatric Inventory in Community-Dwelling Older Adults with Mild Cognitive Impairment and Dementia. J Alzheimers Dis. 2019;68(2):669–678. doi:10.3233/JAD-180641 PMC6538251 30856109

[80] Graham JE, Rockwood K, Beattie BL, Prevalence and severity of cognitive impairment with and without dementia in an elderly population. Lancet. Jun 21 1997;349(9068):1793–6. doi:10.1016/S0140-6736(97)01007-6 9269213

[81] Hyman BT, Phelps CH, Beach TG, National Institute on Aging-Alzheimer’s Association guidelines for the neuropathologic assessment of Alzheimer’s disease. Alzheimers Dement. Jan 2012;8(1):1–13. doi:10.1016/j.jalz.2011.10.007 PMC3266529 22265587

[82] Payne S, Shofer JB, Shutes-David A, Correlates of Conversion from Mild Cognitive Impairment to Dementia with Lewy Bodies: Data from the National Alzheimer’s Coordinating Center. J Alzheimers Dis. 2022;86(4):1643–1654. doi:10.3233/JAD-215428 PMC9536845 35213374

[83] Parkkinen L, Kauppinen T, Pirttila T, Autere JM, Alafuzoff I. Alpha-synuclein pathology does not predict extrapyramidal symptoms or dementia. Ann Neurol. Jan 2005;57(1):82–91. doi:10.1002/ana.20321 15562510

[84] Kovacs GG, Alafuzoff I, Al-Sarraj S, Mixed brain pathologies in dementia: the BrainNet Europe consortium experience. Dement Geriatr Cogn Disord. 2008;26(4):343–50. doi:10.1159/000161560 18849605

[85] Robinson JL, Xie SX, Baer DR, Pathological combinations in neurodegenerative disease are heterogeneous and disease-associated. Brain. Jun 1 2023;146(6):2557–2569. doi:10.1093/brain/awad059 PMC10232273 36864661

[86] Gibson LL, Grinberg LT, Paes VR, Co-pathology in Alzheimer’s disease and Lewy body disease and its association with neuropsychiatric symptoms. Alzheimers Dement. Sep 2025;21(9):e70693. doi:10.1002/alz.70693 PMC12434609 40951924

[87] Clinton LK, Blurton-Jones M, Myczek K, Trojanowski JQ, LaFerla FM. Synergistic Interactions between Abeta, tau, and alpha-synuclein: acceleration of neuropathology and cognitive decline. J Neurosci. May 26 2010;30(21):7281–9. doi:10.1523/JNEUROSCI.0490-10.2010 PMC3308018 20505094

[88] Dhakal S, Wyant CE, George HE, Morgan SE, Rangachari V. Prion-like C-Terminal Domain of TDP-43 and alpha-Synuclein Interact Synergistically to Generate Neurotoxic Hybrid Fibrils. J Mol Biol. May 14 2021;433(10):166953. doi:10.1016/j.jmb.2021.166953 PMC8085152 33771571

[89] Hamilton RL. Lewy bodies in Alzheimer’s disease: a neuropathological review of 145 cases using alpha-synuclein immunohistochemistry. Brain Pathol. Jul 2000;10(3):378–84. doi:10.1111/j.1750-3639.2000.tb00269.x PMC8098522 10885656

[90] Besser LM, Teylan MA, Nelson PT. Limbic Predominant Age-Related TDP-43 Encephalopathy (LATE): Clinical and Neuropathological Associations. J Neuropathol Exp Neurol. Mar 1 2020;79(3):305–313. doi:10.1093/jnen/nlz126 PMC7036651 31845964

[91] Utsumi K, Fukatsu R, Yamada R, Takamaru Y, Hara Y, Yasumura S. Characteristics of initial symptoms and symptoms at diagnosis in probable dementia with Lewy body disease: incidence of symptoms and gender differences. Psychogeriatrics. Sep 2020;20(5):737–745. doi:10.1111/psyg.12586 32743894

[92] Lemstra AW, de Beer MH, Teunissen CE, Concomitant AD pathology affects clinical manifestation and survival in dementia with Lewy bodies. J Neurol Neurosurg Psychiatry. Feb 2017;88(2):113–118. doi:10.1136/jnnp-2016-313775 27794030

[93] Merdes AR, Hansen LA, Jeste DV, Influence of Alzheimer pathology on clinical diagnostic accuracy in dementia with Lewy bodies. Neurology. May 27 2003;60(10):1586–90. doi:10.1212/01.wnl.0000065889.42856.f2 12771246

[94] Gibson LL, Gonzalez MC, Ashton NJ, Plasma phosphorylated tau and neuropsychiatric symptoms in dementia with Lewy bodies. Alzheimers Dement. Feb 2025;21(2):e14434. doi:10.1002/alz.14434 PMC11848415 39732510

[95] Barnes LL, Lamar M, Schneider JA. Sex differences in mixed neuropathologies in community-dwelling older adults. Brain Res. Sep 15 2019;1719:11–16. doi:10.1016/j.brainres.2019.05.028 PMC6636678 31128096

[96] Horsager J, Borghammer P. Brain-first vs. body-first Parkinson’s disease: An update on recent evidence. Parkinsonism Relat Disord. May 2024;122:106101. doi:10.1016/j.parkreldis.2024.106101 38519273

[97] Thomas AJ, Mahin-Babaei F, Saidi M, Improving the identification of dementia with Lewy bodies in the context of an Alzheimer’s-type dementia. Alzheimers Res Ther. Mar 1 2018;10(1):27. doi:10.1186/s13195-018-0356-0 PMC5831205 29490691

[98] Rizzo G, Arcuti S, Copetti M, Accuracy of clinical diagnosis of dementia with Lewy bodies: a systematic review and meta-analysis. J Neurol Neurosurg Psychiatry. Apr 2018;89(4):358–366. doi:10.1136/jnnp-2017-316844 29030419

[99] Singh PP, Singh M, Mastana SS. APOE distribution in world populations with new data from India and the UK. Ann Hum Biol. May-Jun 2006;33(3):279–308. doi:10.1080/03014460600594513 17092867

[100] Filshtein TJ, Dugger BN, Jin LW, Neuropathological Diagnoses of Demented Hispanic, Black, and Non-Hispanic White Decedents Seen at an Alzheimer’s Disease Center. J Alzheimers Dis. 2019;68(1):145–158. doi:10.3233/JAD-180992 PMC7286069 30775996

