# Supplementary material for: The prevalence of Lewy body pathology across nine international community-based cohorts
Source: Brain. Author manuscript; Available in PMC 2026 Oct 17. (PMC13675927; doi:10.1093/brain/awag112)
Supplement: Supplementary file 1 [file NIHMS2209851-supplement-Additional_supplemental_material__1-11_.pdf]

Supplementary table 1. Cohorts included with select site-specific methodologies

**Cohort name**	**Country**	**Age range**	**Population**	**Lewy body pathology (LBP) staging criteria**	**Year of study start**	**Post-mortem interval (mean hours)**	**Alpha synuclein antibody used**	**TDP-43 antibody used**	**Assessment of cognitive status**
Adult Changes in Thought (ACT)[1]	USA	65+	Kaiser Permanente members, Seattle area	McKeith staging (including olfactory only)[2]	1994	16.6	LB509	1D3 antibody (anti-pTDP-43 [pS409/pS410])	DSM-IV dementia diagnosis. CASI score to divide non-dementia group into high vs low cognitive function
BAS-USP[3-5]	Sao Paulo, Brazil	50+	General city population, Sao Paulo, Brazil	McKeith staging[2]	2004	14.5	Purified anti-α-Synuclein Phospho (Ser129) Antibody	1D3 antibody (anti-pTDP-43 [pS409/pS410])	Established from CDR conducted post-mortem from NOK.[6,7]
Baltimore Longitudinal Study of Aging [8,9]	USA	60+	Community cohort in Baltimore, Maryland.	McKeith staging[2]	1986	20.4	Clon 42	anti-pTDP-43 [pS409/pS410]	No information on cognitive status provided.
Duke-UNC ADRC[10]	USA	45+	Community cohort in North Carolina.	McKeith staging[2]	1985	16.2	BD Biosciences mouse anti-alpha-synuclein Cat# 610787	c-terminal TDP-43 antibody from Proteintech (Cat# 12892-1-AP).	Dementia diagnosis made via consensus conference supported by CDR (cognitively normal: 0 in CDR and normal cognitive testing; MCI: impaired memory scores and function preserved equalling 0.5 CDR; dementia: if CDR>0.5 with impairment to memory and a second cognitive domain).

ROS-MAP [11,12]	USA	65+	Older persons without known dementia from communities across Greater Chicago and Catholic nuns, priests and brothers from 40 sites across the US.	McKeith staging (neocortical, limbic and brainstem) [13,14]	1997	7.3 (median)	Anti phosphorylated alpha –synuclein (SER129) Monoclonal antibody with alkaline phosphatase as the chromogen	1D3 antibody (anti-pTDP-43 [pS409/pS410]	Dementia diagnoses made by a clinician according to established criteria.[15-17] MCI identified if cognitive impairment established by neuropsychologist in absence of clinical diagnosis of dementia made by examining clinician.
UCI 90+ study [18-20]	USA	90+	Community cohort in Orange County, California.	Braak staging[21]	2003	8.5	phospho S129	1D3 antibody (anti-pTDP-43 [pS409/pS410])	DSM-IV dementia diagnosis made by consensus conference
UK-UNC ADRC [22]	USA	60+	Community cohort in Fayette County, Kentucky.	McKeith staging (including olfactory only)[2]	1989	7.1	KM51 Clone, Leica	1D3 antibody (anti-pTDP-43 [pS409/pS410])	Dementia diagnosis was established in consensus conference based on NINCDS-ADRDA criteria.[15] MCI was classified using published criteria.[23]
Vantaa 85+[24]	Finland	85+	General city population, Vantaa, South Finland	McKeith staging (including olfactory only)[2]	1991	64.2	5G4	anti-C-terminal-TDP-43 antibody (Proteintech)	DSM-IIIR criteria were used to diagnose dementia. No MCI diagnosis.
VITA[25-27]	Austria	Born 1925-6	Population-based cohort from Vienna district	McKeith staging[2]	2000	Not available	5G4 epitope 47-53, PMID 22370907, Roboscreen GmbH Leipzig, Germany	anti-phospho-TDP-43 (pS409/410, 1:2,000, Cosmo Bio, Tokyo, Japan),—the same clone 1D3.	No information on cognitive status provided.

Abbreviations: CASI, Cognitive Abilities Screening Instrument; CDR, clinical dementia rating; DSM, Diagnostic and Statistical Manual of Mental Disorders; MCI, mild cognitive impairment; NOK, next of kin.

Supplementary Table 2. Supplementary information on included cohorts

Cohorts (in alphabetical order)	Notes	Ref(s)
Adult Changes in Thought (ACT)	A population-based prospective cohort study investigating brain aging and dementia risk factors within Kaiser Permanente Washington (formerly Group Health Cooperative), an integrated health care delivery system in Washington State. Community-dwelling, adults aged 65 years and older without dementia are recruited from among Kaiser Permanente members residing in the Seattle area. The demographic composition reflects the surrounding county in which a majority of people are white and relatively well-educated. Consent for brain donation is not required at enrolment; however, brain autopsy consent is consistently obtained from approximately 25–30% of participants, with rates increasing in recent years. At study entry and every two years thereafter, participants undergo cognitive screening, physical function testing, medical history review, and assessments of functional status.	1
Baltimore Longitudinal Study of Aging (BLSA)	Longitudinal study of normal aging with subjects 65 years or older enrolled in a prospective autopsy program in Baltimore, Maryland. The study was initiated in 1958 with recruitment initially restricted to men; women were included from 1978. The autopsy program was established in 1986. The cohort is predominantly male, white, and highly educated. However, no clinical or demographic data accompanied the neuropathological data from the BLSA included in this study.	8,9
Brazilian Biobank for Aging Studies from University Sao Paulo (BAS-USP)	Population-based autopsy study with subjects who died from non-forensic causes of death in the city of Sao Paulo, Brazil. Participants were 50 years or older, with diverse ethnic composition similar to the overall death data from São Paulo. The study has been ongoing since 2003, and all the donations are sourced from the Autopsy Service of the University of Sao Paulo, the only service providing non-forensic autopsies (around 15,000 per year) in this city of over 11 million inhabitants. 16% of the annual deaths in the city of São Paulo are autopsied at the Autopsy Service of the University of São Paulo. In BAS cases with inconsistent clinical information, postmortem interval >24 hours, or poor tissue quality (e.g., cerebrospinal fluid pH <6.5 or major acute brain lesions) were excluded.	3-5
Duke-UNC Alzheimer's Disease Research Center (Duke ADRC)	Participants over 90 years of age at death were included in this study. Approximately 70% of the cohort had normal cognition at the time of recruitment. More than 95% of participants are White. Consensus meetings were held yearly to review and update the clinical diagnosis, based upon contemporaneous NIA-AA criteria.	28
Rush Religious Orders Study – Memory and Aging Project (ROS-MAP)	Both are Rush University ongoing epidemiologic longitudinal clinical-pathologic studies of older persons. The Religious Order Study (ROS) began in 1994 and recruits older priests, nuns, and brothers from 45 sites across the United States. The Rush Memory and Aging Project (MAP) began in 1997 and recruits participants from across the Chicagoland metropolitan area. In both studies, participants were enrolled at age 65 and above without known dementia and agreed to undergo annual clinical evaluation and interview, including detailed neuropsychological testing, and brain donation. One third had 12 or fewer years of education and 10% were members of an ethnic or racial minority group.	11,12
The 90+ Study	Community-based autopsy cohort in Southern California, USA. Participants in The 90+ Study were initially survivors of the Leisure World Cohort Study (LWCS), a longitudinal epidemiological investigation of a retirement community in Orange County, CA, established in the early 1980s. The 90+ Study began in 2003, when surviving LWCS participants aged 90 years and older as of January 1, 2003 were invited to enroll. Additional invitations were extended on January 1, 2008, and annually thereafter, to LWCS participants on reaching age 90. More recently, open recruitment of volunteers aged 90 and older outside of LWCS was initiated through multiple strategies including residential mailing lists, community talks, outreach to primary care physicians, local newspaper advertisements, and referrals relatives or friends of participants. LWCS participants were recruited irrespective of cognitive status, whereas non-LWCS volunteers were limited to those with no or mild dementia. Approximately two-thirds of participants are from the original LWCS, and one-third are non-LWCS volunteers. The cohort is predominantly White (98%) and 50% are college educated.	19,20

U. Kentucky Alzheimer's Disease Research Center (Uky ADRC)	Community-based autopsy cohort in Lexington, Kentucky based on an NIH-funded ADRC. Recruitment began in the mid-1980's, enrolling cognitively normal adults aged 70 years and older who were followed annually until death. Final diagnosis prior to death refers to the diagnosis of a consensus group including clinicians and pathologists, whereas the PDD or DLB clinical syndrome was evaluated by a neurologist during life. More than 95% of included autopsied participants were White/Caucasian. In 2012 the autopsy rate since inception was 75%, increasing to 85% for persons consented after 2005. Mean years of education in autopsied patients 16.0+2.5.	29
Vantaa 85+	A population-based study, which includes all the subjects who lived in the city of Vantaa, Finland, in 1991 and were 85 years or over. > 50% of the population were neuropathologically examined.	24
Vienna Trans-Danube Aging (VITA)	Community-based autopsy cohort in Vienna, Austria. The VITA study was established in 2000 and enrolled all residents of Vienna's 21st and 22nd districts born between May 1925 and June 1926. Participants were recruited at age 75 and followed longitudinally.	25-27

Supplementary figure 1. Random effects meta-analysis of prevalence of neocortical LBP across community cohorts grouped by cognitive status.

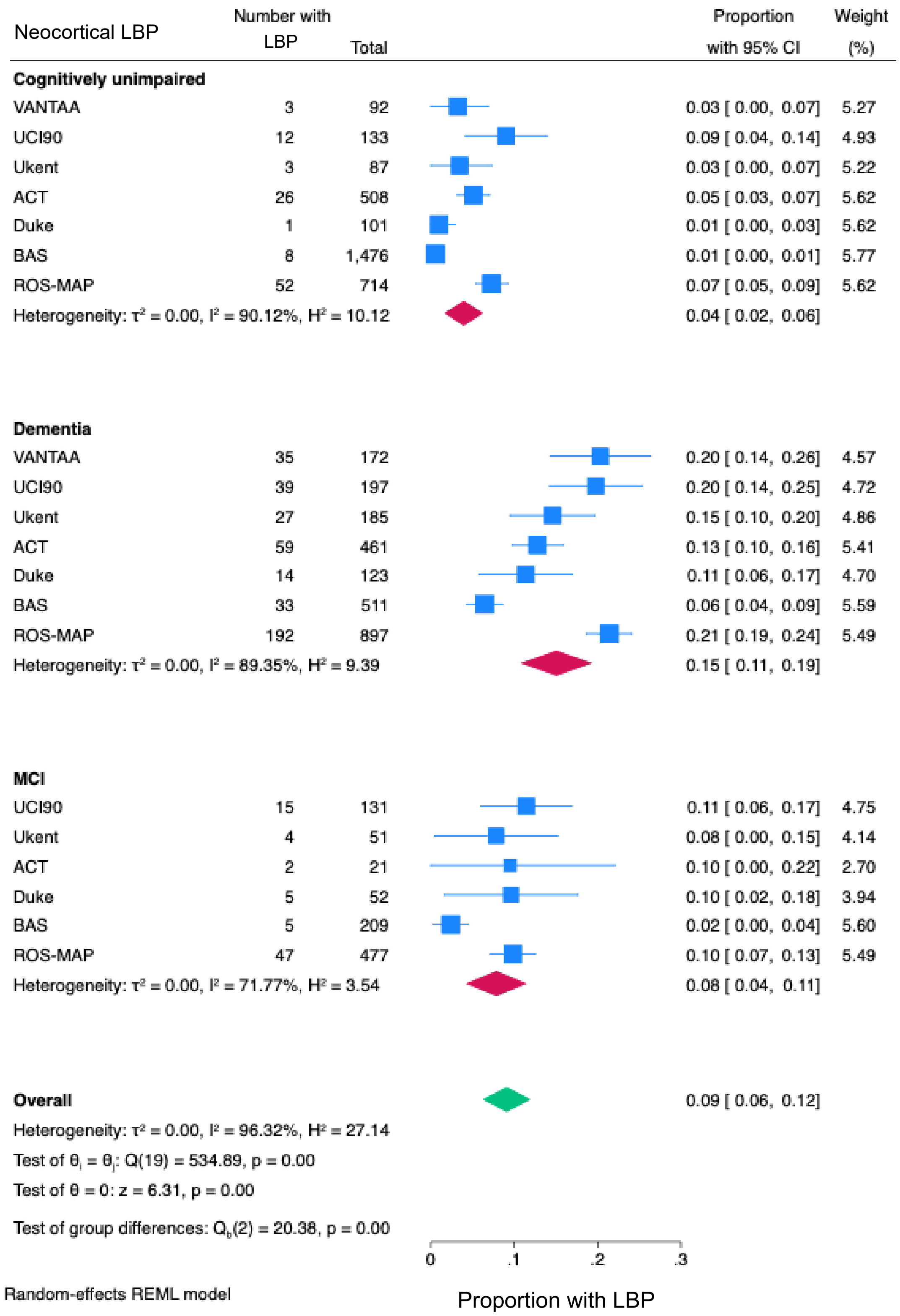

Supplementary figure 2. Random effects meta-analysis of prevalence of limbic-predominant LBP across community cohorts grouped by cognitive status.

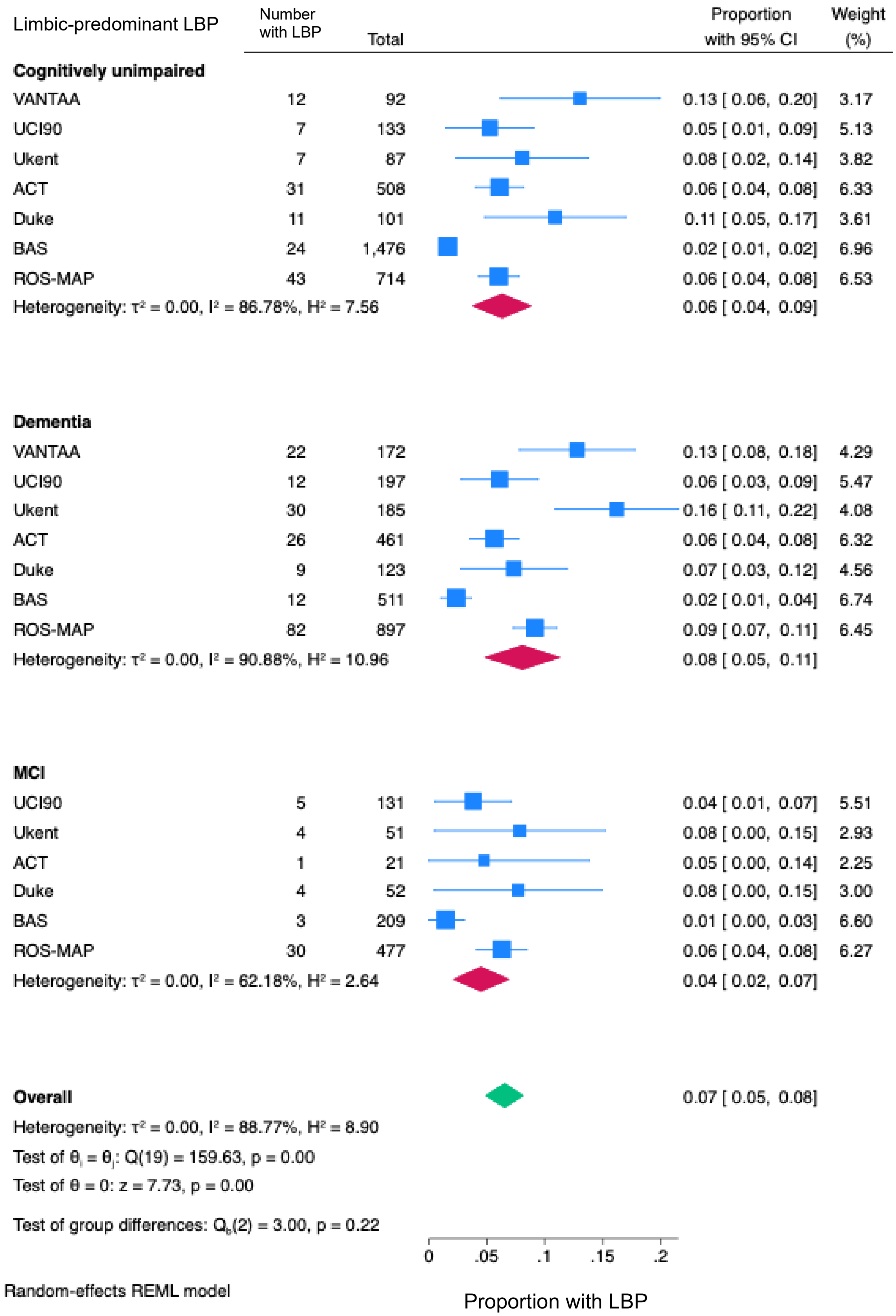

Supplementary figure 3. Random effects meta-analysis of prevalence of brainstem-predominant LBP across community cohorts grouped by cognitive status.

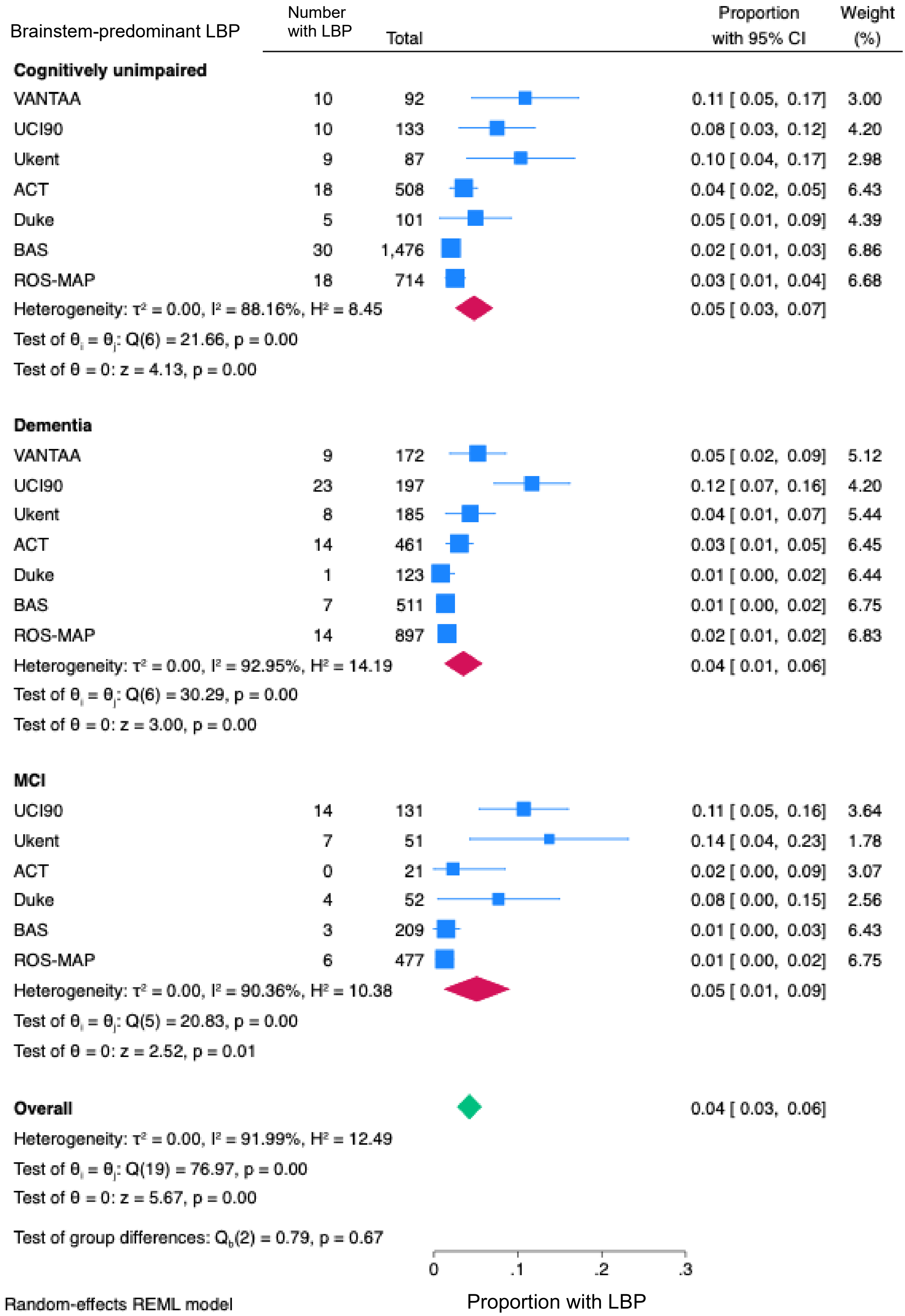

Supplementary figure 4. Random effects meta-analysis of prevalence of amygdala-predominant LBP across community cohorts grouped by cognitive status.

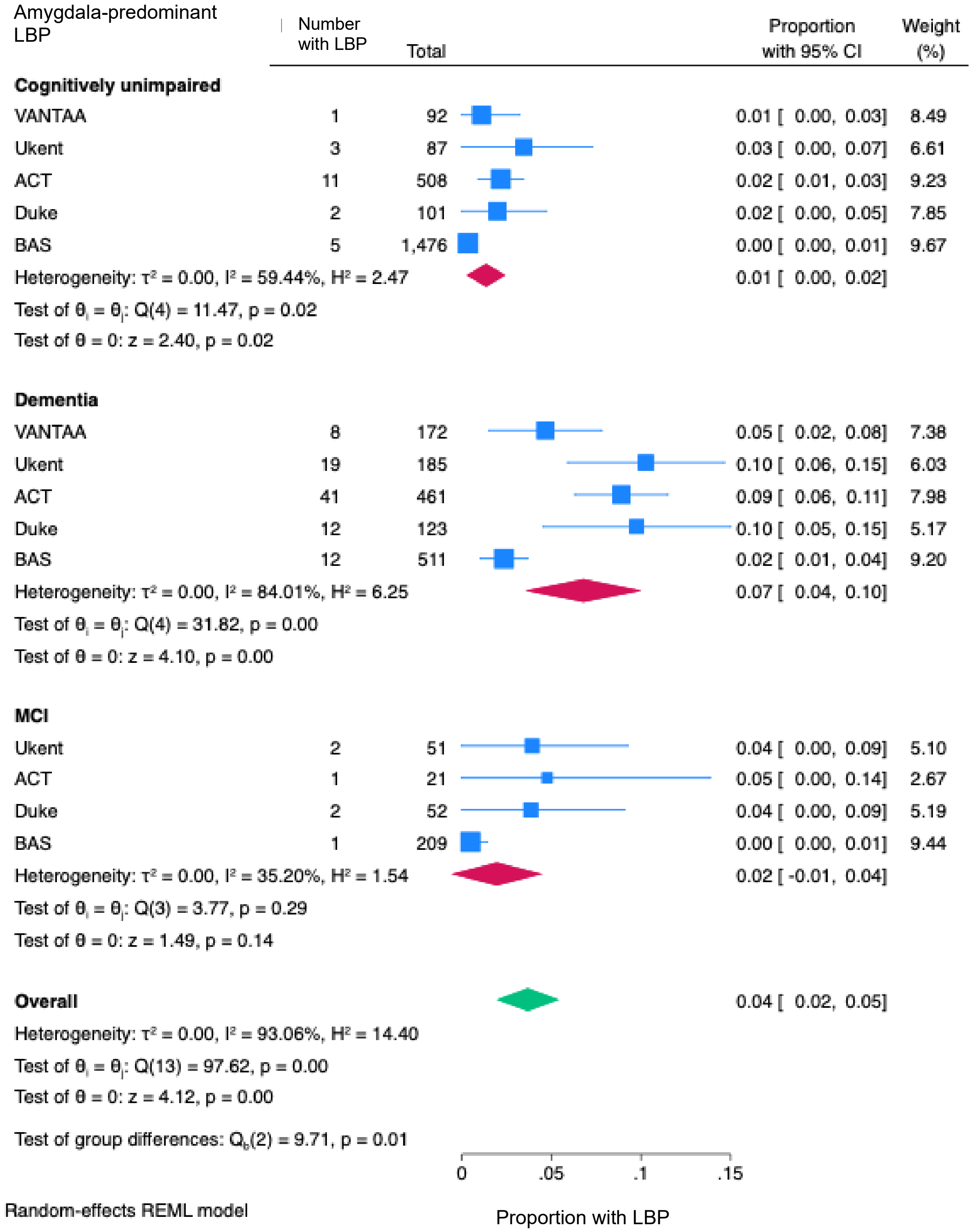

Supplementary figure 5. Random effects meta-analysis of prevalence of participants with each staging of NFT across cohorts for individuals with neocortical LBP.

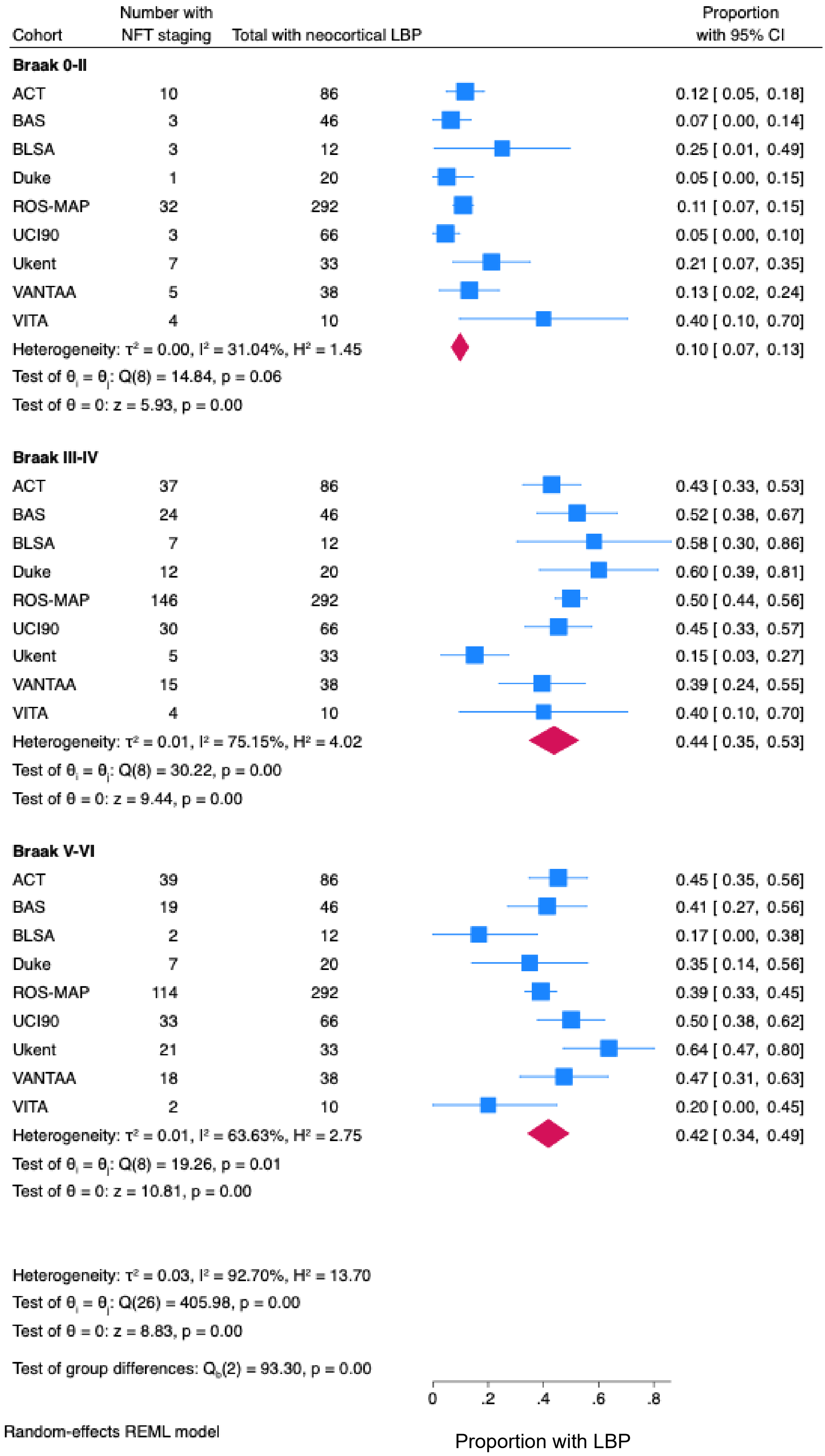

Supplementary figure 6. Random effects meta-analysis of prevalence of participants with each staging of NFT across cohorts in individuals with limbic-predominant LBP.

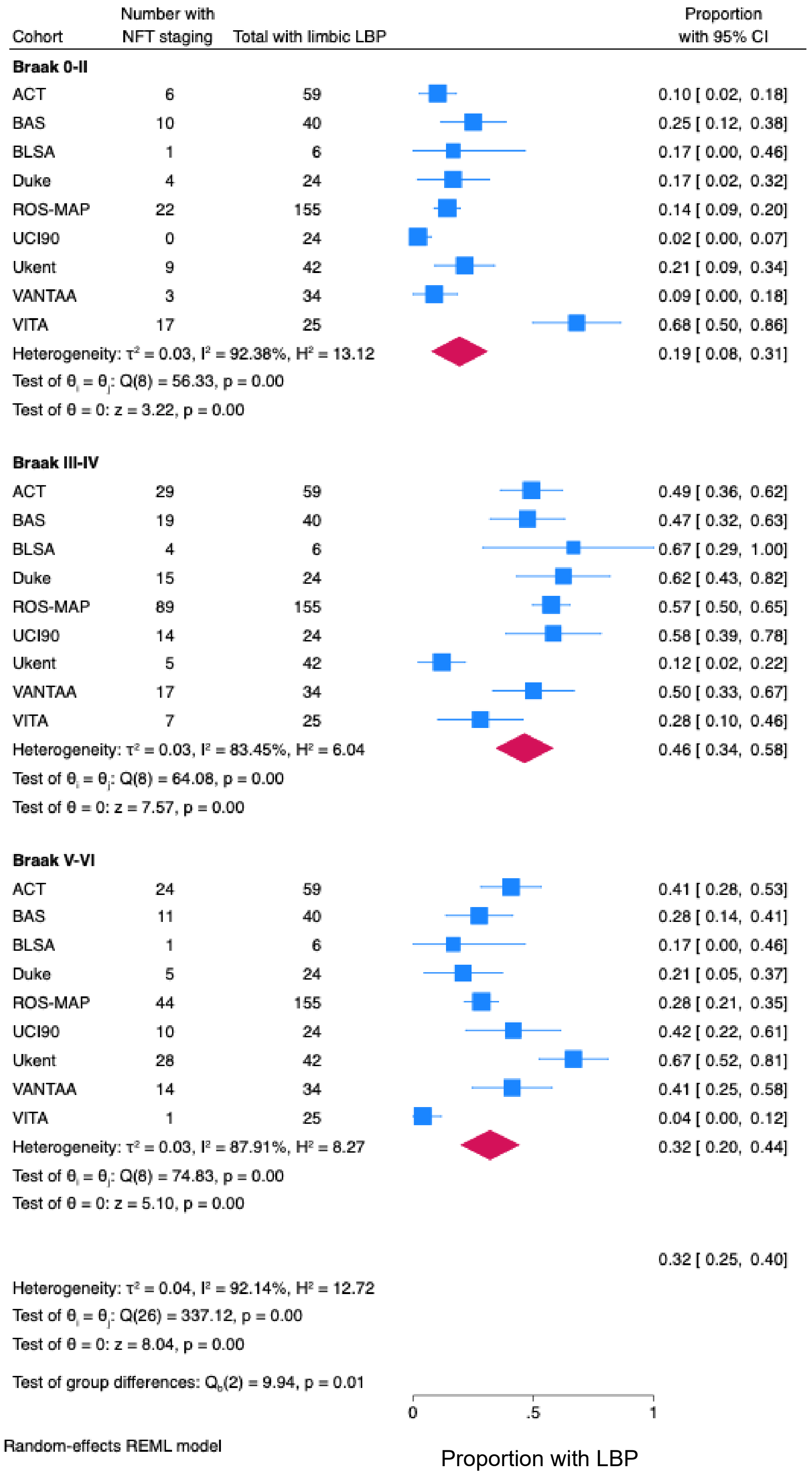

Supplementary figure 7. Random effects meta-analysis of prevalence of participants with each staging of NFT across cohorts in individuals with brainstem-predominant LBP.

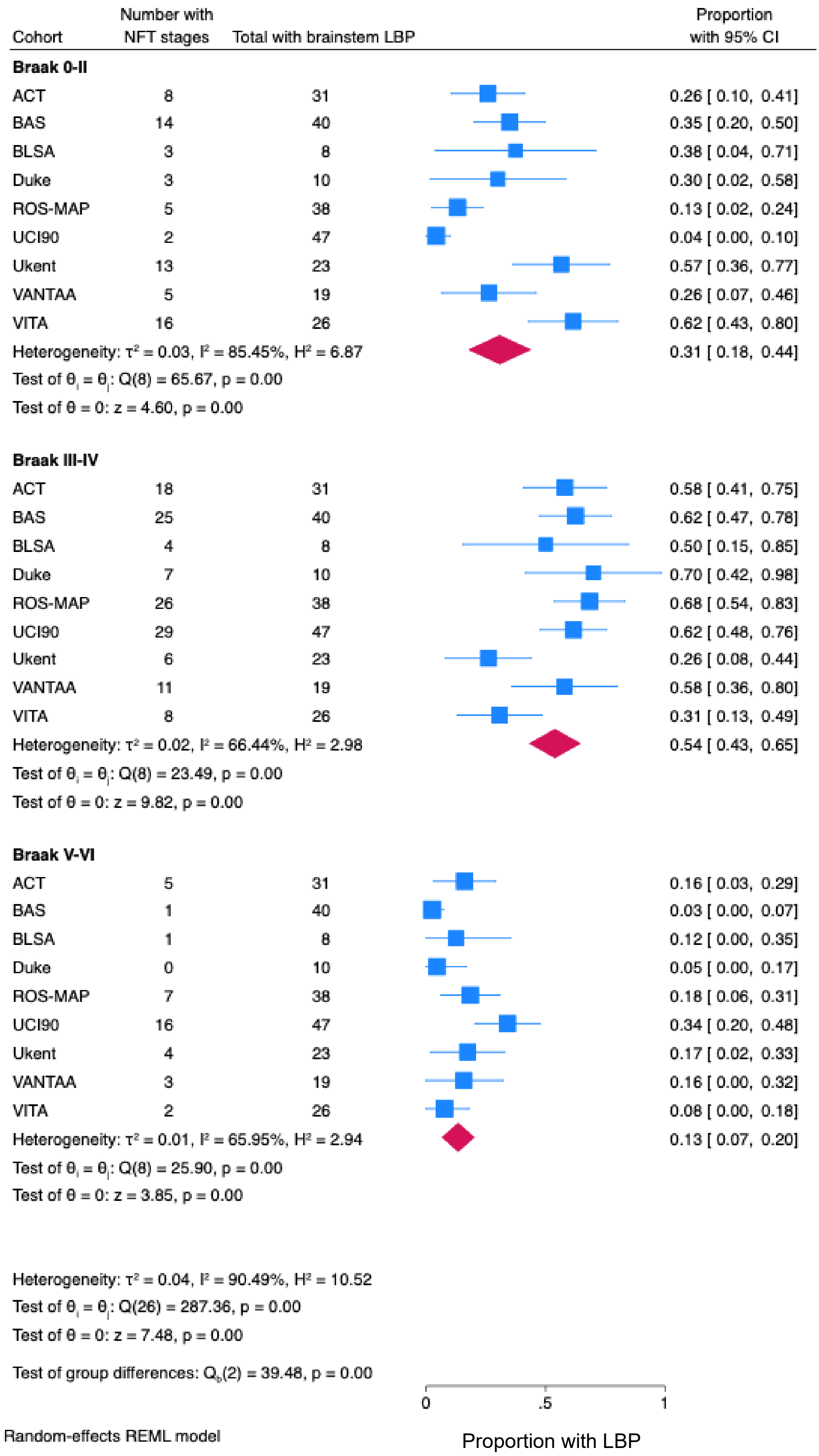

Supplementary figure 8. Random effects meta-analysis of prevalence of participants with each staging of NFT across cohorts in individuals with amygdala-predominant LBP.

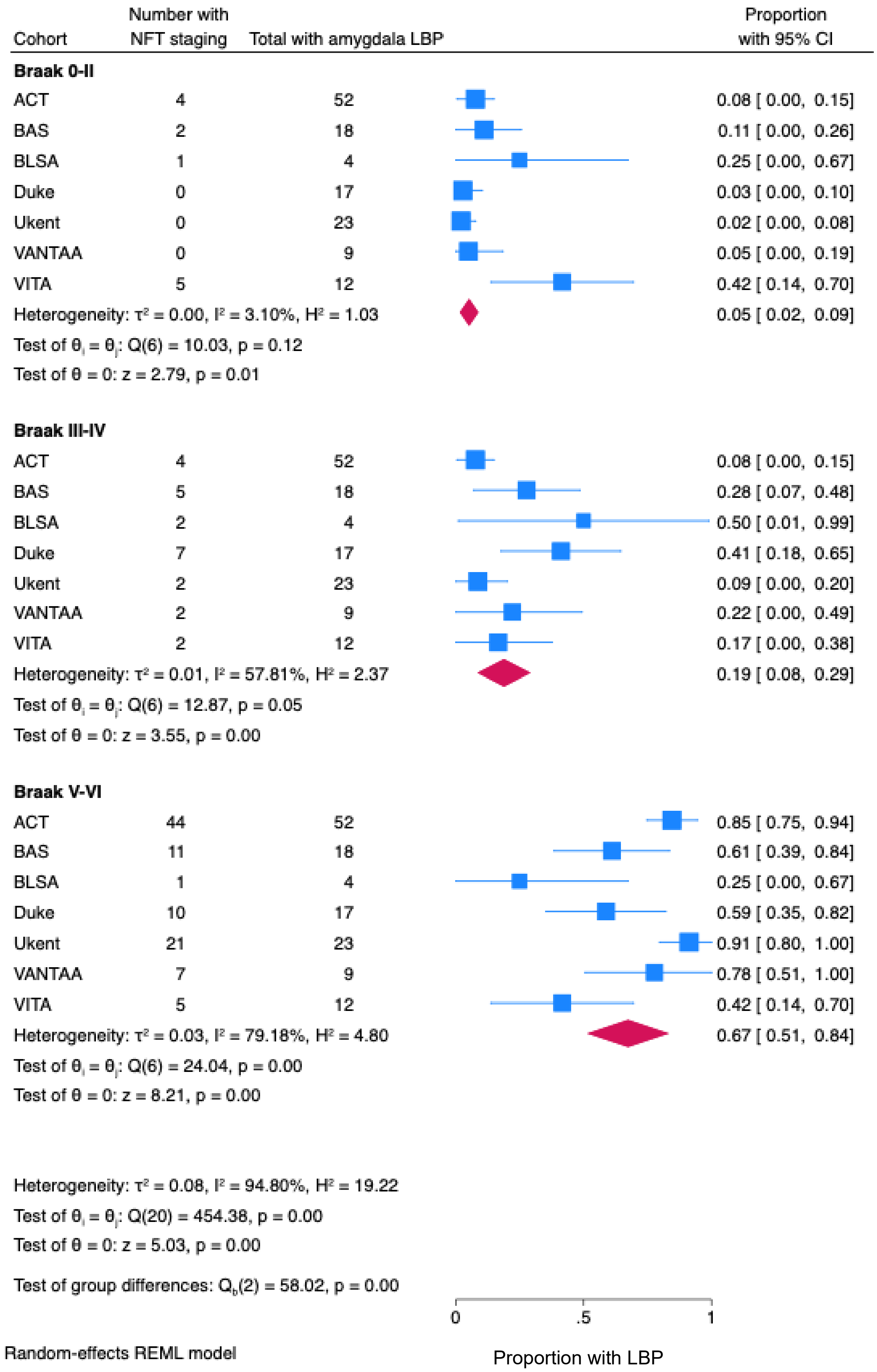

Supplementary figure 9. Scatter graphs showing mean age of cohort with prevalence of each subtype of LBP with spearman correlation.

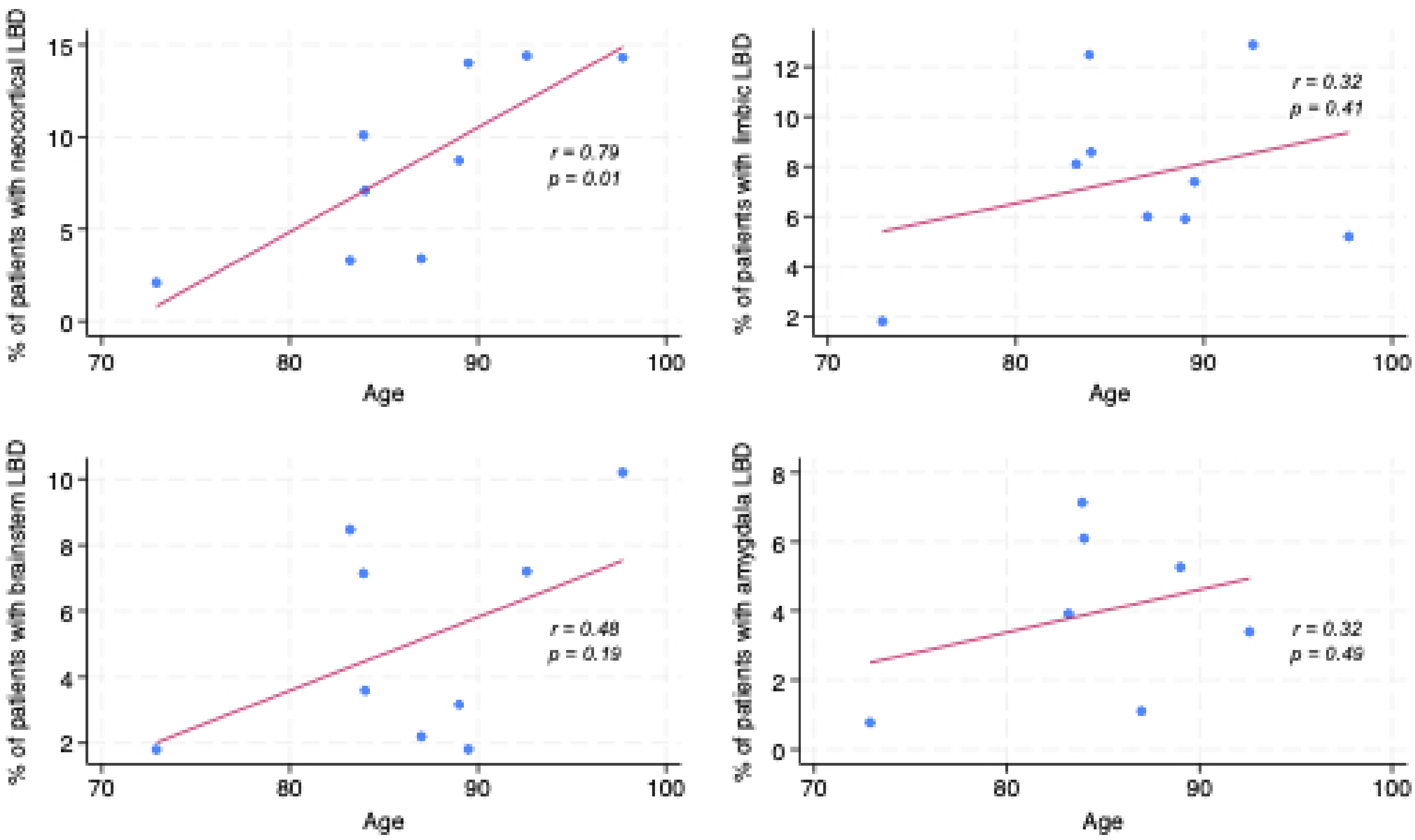

Supplementary figure 10. Scatter graphs showing spearman correlation between percentage with APOE allele 4 positivity and % of patients with each subtype of LBP for each cohort. No significant correlation was detected.

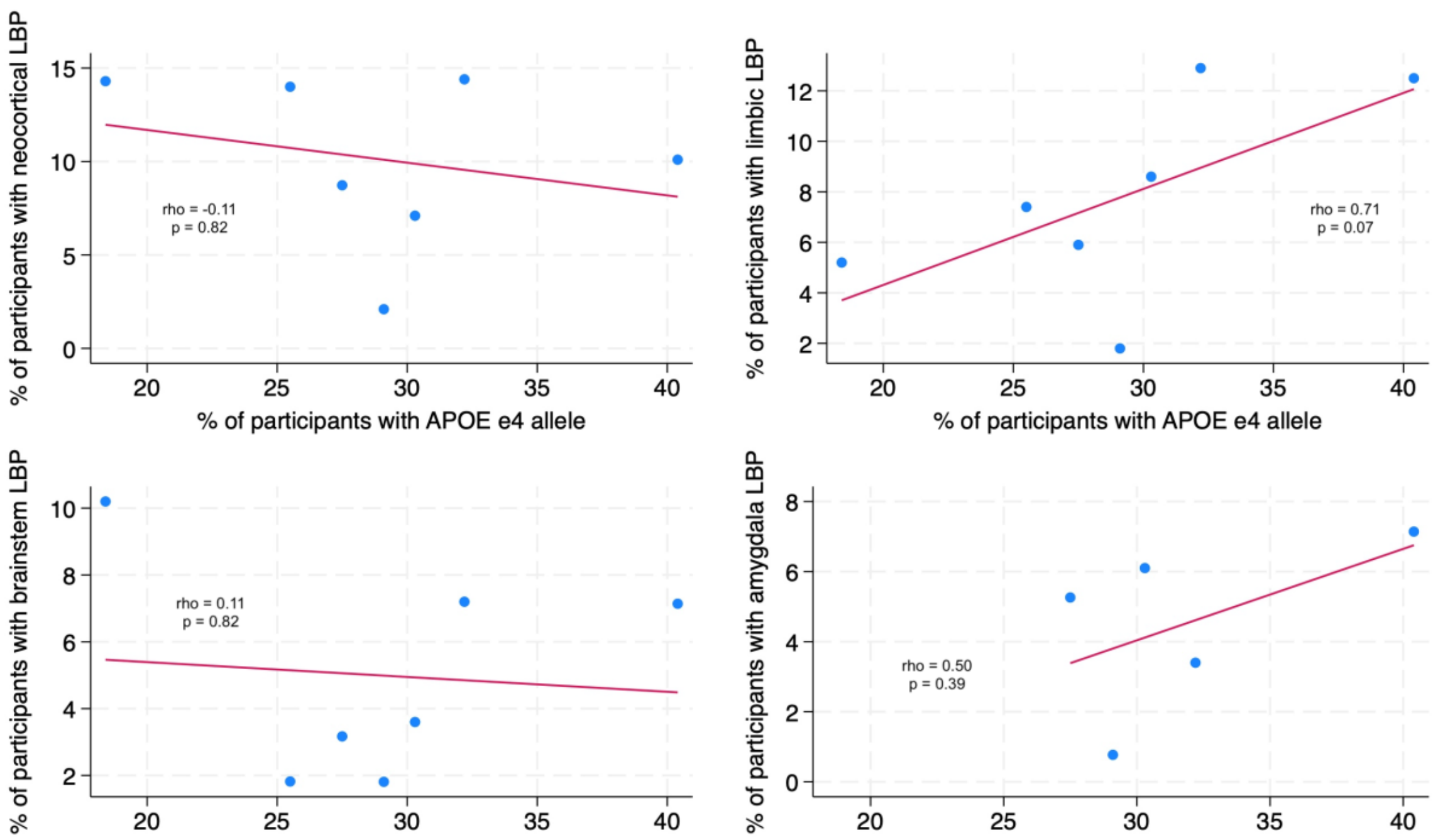

Supplementary figure 11. Random effects meta-analysis of prevalence of neocortical LBP across community cohorts grouped by sex.

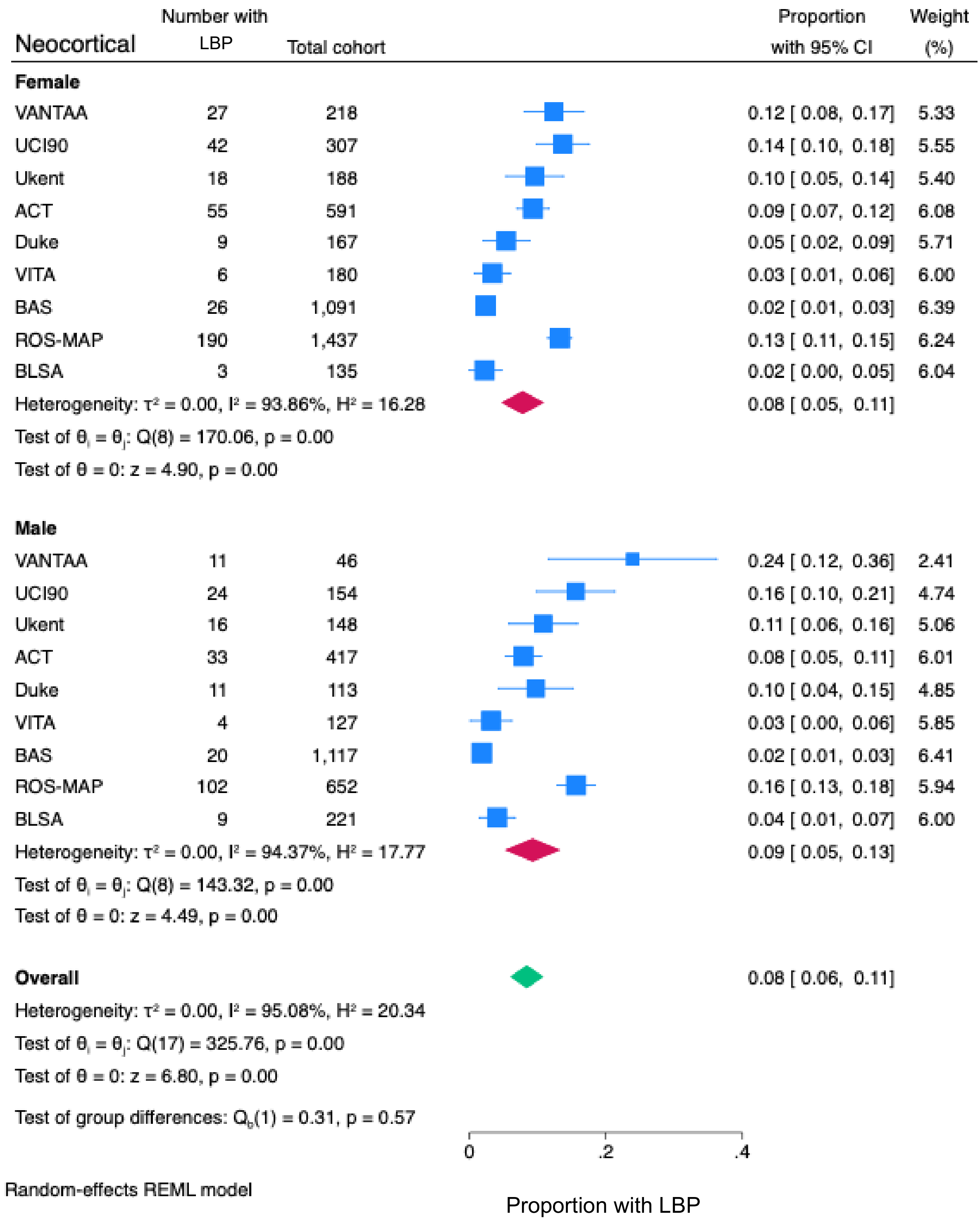

Supplementary figure 12. Random effects meta-analysis of prevalence of limbic-predominant LBP across community cohorts grouped by sex.

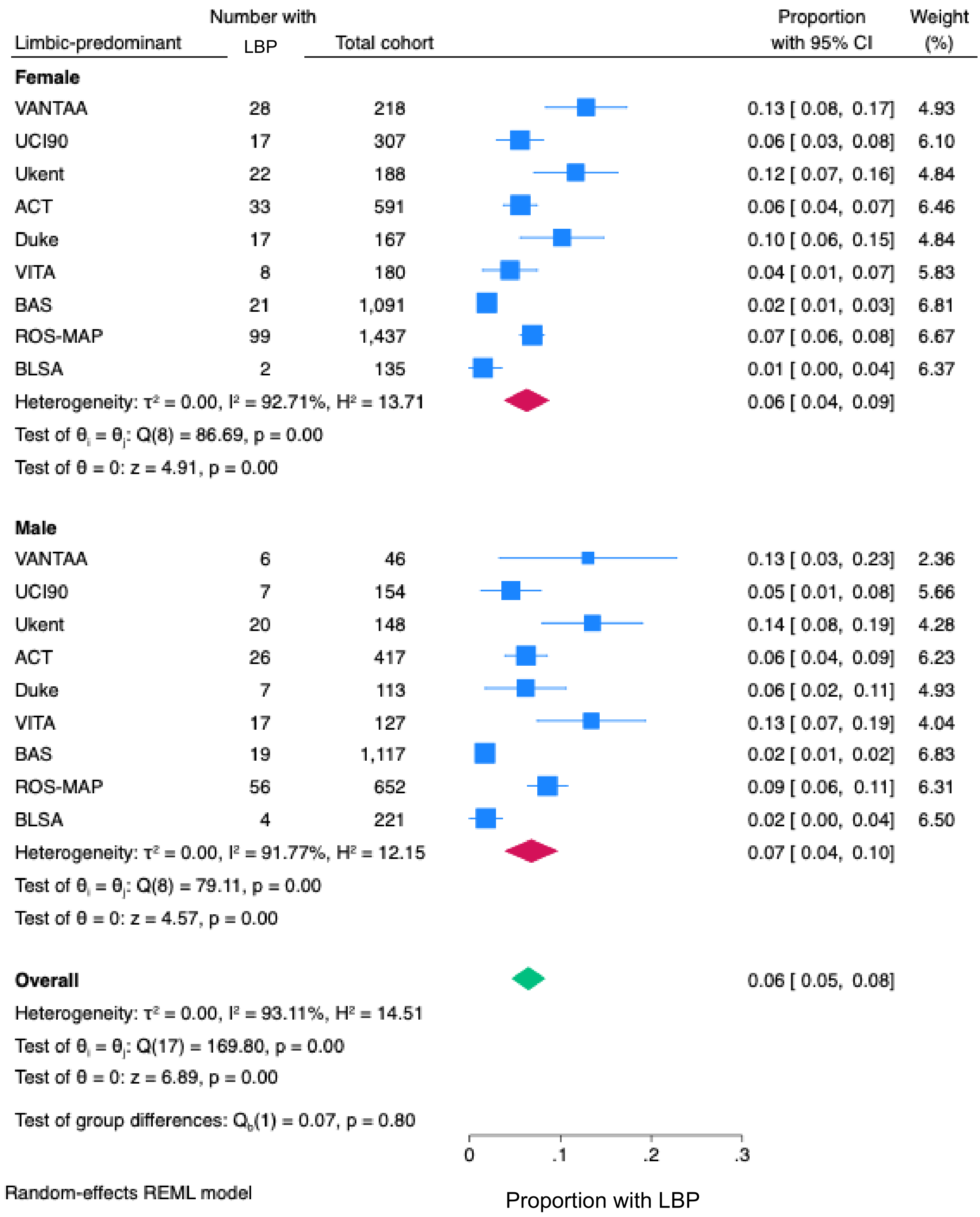

Supplementary figure 13. Random effects meta-analysis of prevalence of brainstem-predominant LBP across community cohorts grouped by sex.

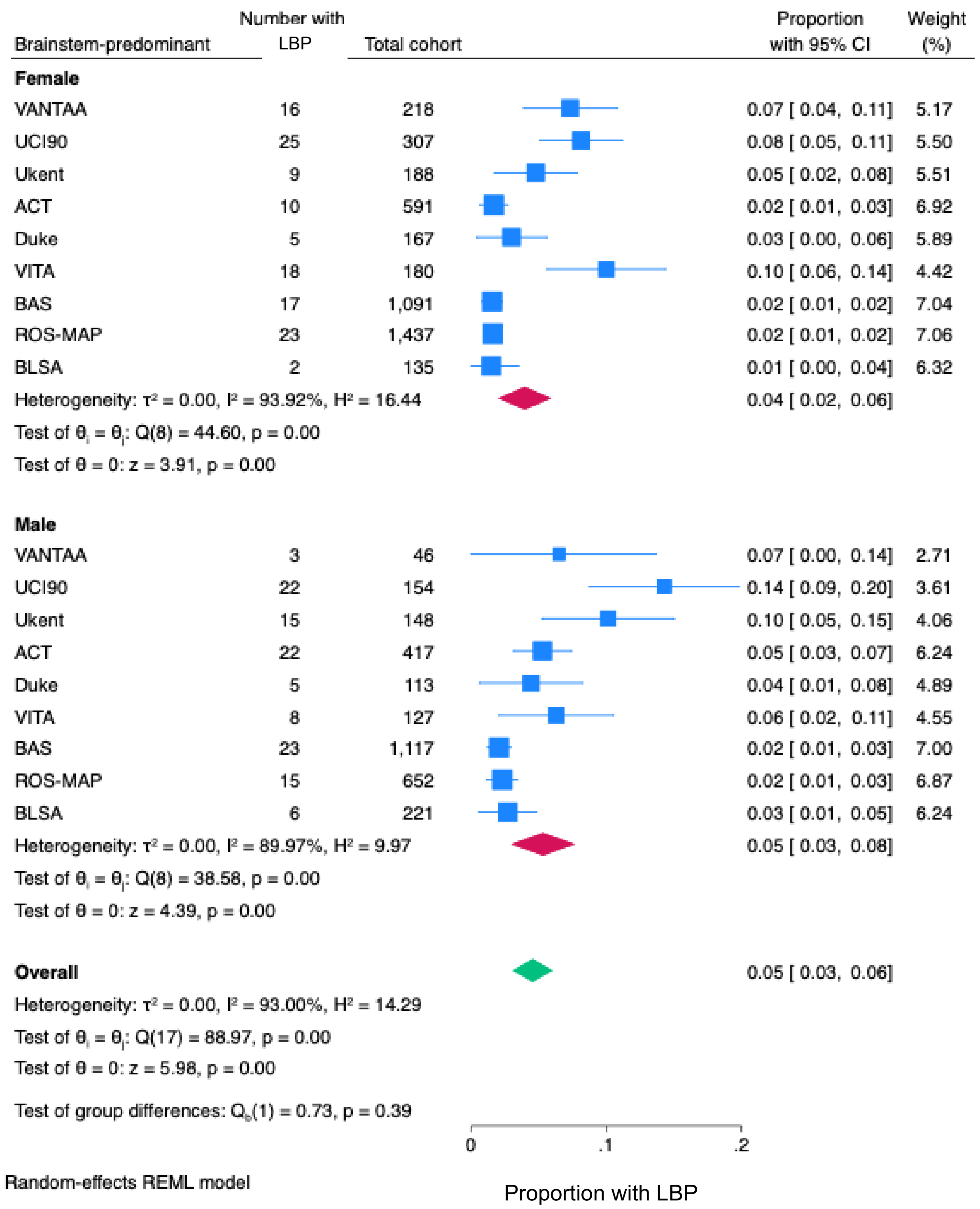

Supplementary figure 14. Random effects meta-analysis of prevalence of amygdala-predominant LBP across community cohorts grouped by sex.

Amygdala-predominant	Number with LBP	Total cohort	Proportion with 95% CI	Weight (%)
**Female**				
VANTAA	8	218	0.04 [0.01, 0.06]	7.41
Ukent	13	188	0.07 [0.03, 0.11]	5.71
ACT	37	591	0.06 [0.04, 0.08]	8.26
Duke	9	167	0.05 [0.02, 0.09]	6.00
VITA	7	180	0.04 [0.01, 0.07]	6.89
BAS	15	1,091	0.01 [0.01, 0.02]	9.83
BLSA	3	135	0.02 [0.00, 0.05]	7.42
Heterogeneity: $\tau^2 = 0.00$, $I^2 = 75.59\%$, $H^2 = 4.10$			0.04 [0.02, 0.06]	
Test of $\theta_i = \theta_j$: $Q(6) = 34.39$, $p = 0.00$				
Test of $\theta = 0$: $z = 4.71$, $p = 0.00$				
**Male**				
VANTAA	1	46	0.02 [0.00, 0.06]	4.96
Ukent	11	148	0.07 [0.03, 0.12]	4.95
ACT	16	417	0.04 [0.02, 0.06]	8.43
Duke	8	113	0.07 [0.02, 0.12]	4.38
VITA	5	127	0.04 [0.01, 0.07]	6.06
BAS	3	1,117	0.00 [0.00, 0.01]	10.05
BLSA	1	221	0.00 [0.00, 0.01]	9.66
Heterogeneity: $\tau^2 = 0.00$, $I^2 = 92.27\%$, $H^2 = 12.93$			0.03 [0.01, 0.05]	
Test of $\theta_i = \theta_j$: $Q(6) = 37.10$, $p = 0.00$				
Test of $\theta = 0$: $z = 2.86$, $p = 0.00$				
**Overall**			0.03 [0.02, 0.05]	
Heterogeneity: $\tau^2 = 0.00$, $I^2 = 91.03\%$, $H^2 = 11.15$				
Test of $\theta_i = \theta_j$: $Q(13) = 104.16$, $p = 0.00$				
Test of $\theta = 0$: $z = 5.20$, $p = 0.00$				
Test of group differences: $Q_b(1) = 0.48$, $p = 0.49$				

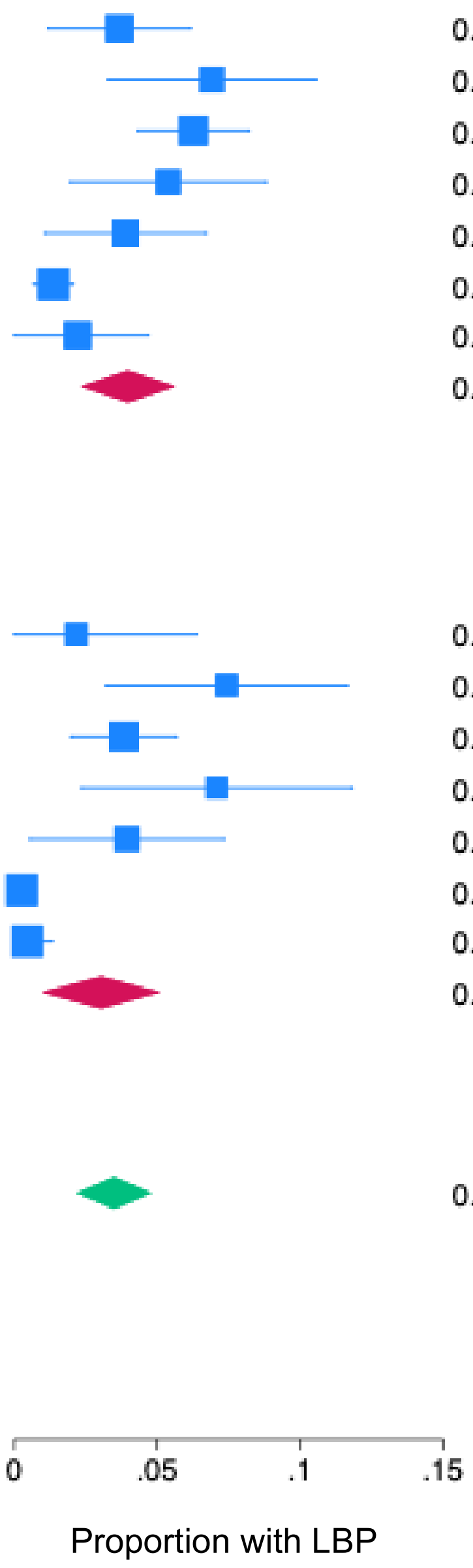

Random-effects REML model

Supplementary figure 15. Odds ratio for each subtype of LBP in females versus males

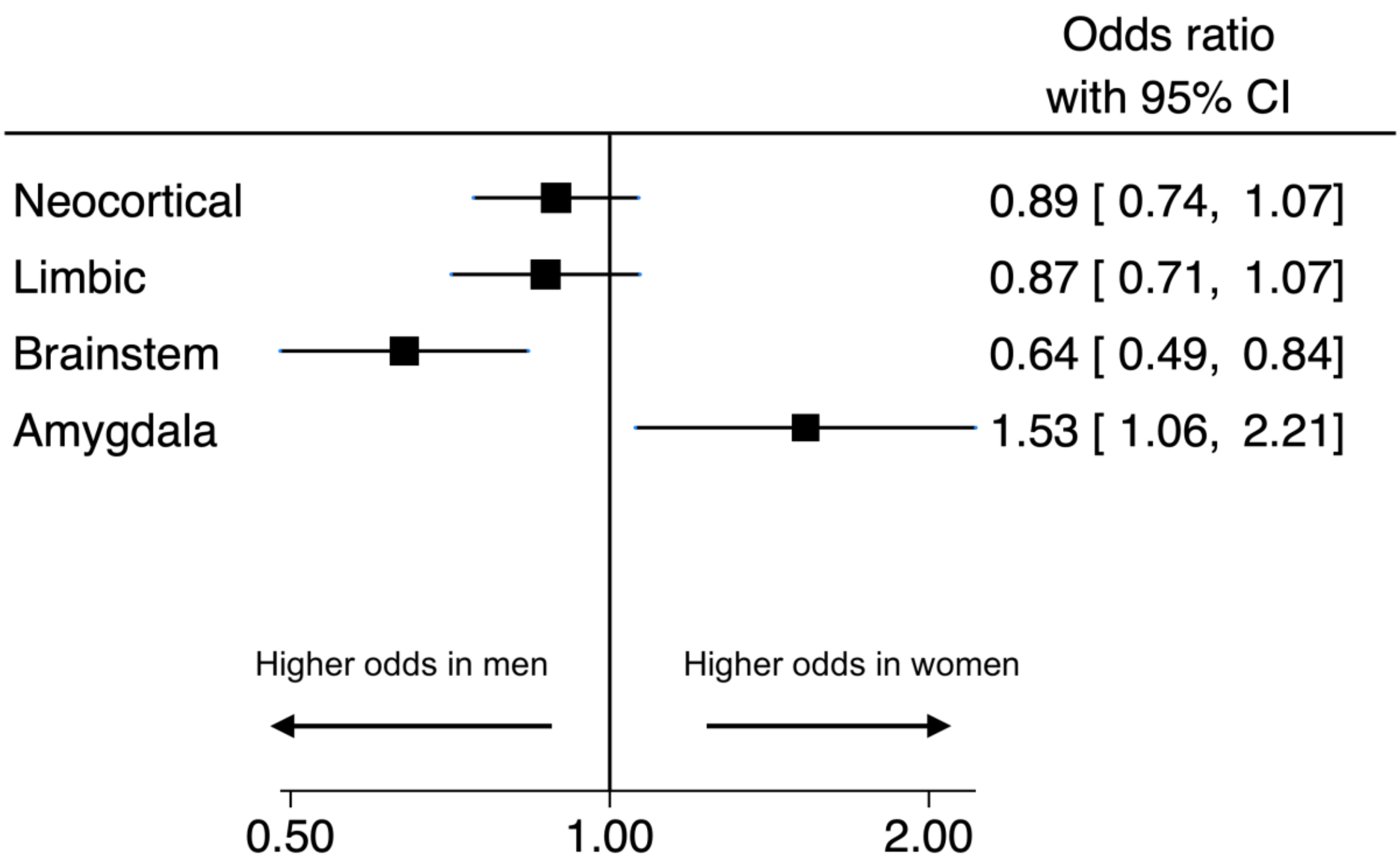

Supplementary table 3. Subgroup analysis from three cohorts (BAS, ROS-MAP, UK) showing prevalence of ADNC in each subtype of LBP stratified by sex.

	All participants across 3 cohorts		Number with ADNC co-pathology			
LBP subtype	Men (n=1547)	Women (n=2237)	Men	Women	X^2	p value
Neocortical (n=364)	132	232	90 (68.2%)	175 (75.4%)	2.23	0.14
Limbic (n=232)	90	142	52 (57.8%)	96 (67.6%)	2.30	0.13
Brainstem (n=105)	57	48	20 (35.1%)	16 (33.3%)	0.04	0.85
Amygdala[a] (n=49)	14	35	12 (85.7%)	32 (91.4%)	0.35	0.55
Olfactory only[b] (n=18)	12	6	10 (83.3%)	5 (83.3%)	0.00	1.00
No LBP (n=3633)	1495	2138	467 (31.2%)	1053 (49.2%	117.3	<0.001

Difference in proportions across sex were evaluated in χ^2 tests. Significant results highlighted in bold if $p<0.05$.
[a]reported only in UKy and BAS, [b]reported only in UKy

Supplementary table 4. Subgroup analysis from three cohorts (Duke, UCI90 and UK) showing neuropathological staging for participants with clinical diagnoses of DLB and PDD.

**Neuropathologic features**	**DLB n=29**	**PDD n=11**	**p value**
Neocortical LBP, n (%)	12 (41.3)	5 (45.5)	0.82
Limbic LBP, n (%)	2 (7.9)	4 (36.4)	0.039
Brainstem LBP, n (%)	5 (17.2)	1 (9.1)	1
None, n (%)	10 (34.5)	1 (9.1)	0.233
Thal Aβ phases 4 or 5, n (%)	19 (65.5)	7 (63.6)	1
CERAD moderate or frequent, n (%)	21 (72.4)	5 (45.5)	0.147
Braak NFT stages V-VI, n (%)	16 (55.2)	3 (27.3)	0.163
LATE NC stage 2 or 3, n (%)	6 (20.7)	2 (18.2)	1

Difference in proportions across sex were evaluated in χ^2 tests or fisher's exact where cell count was less than 5. Significant results highlighted in bold if p<0.05.

Supplementary figure 16. Percentage of participants with core features of DLB for each anatomical distribution of LBP.

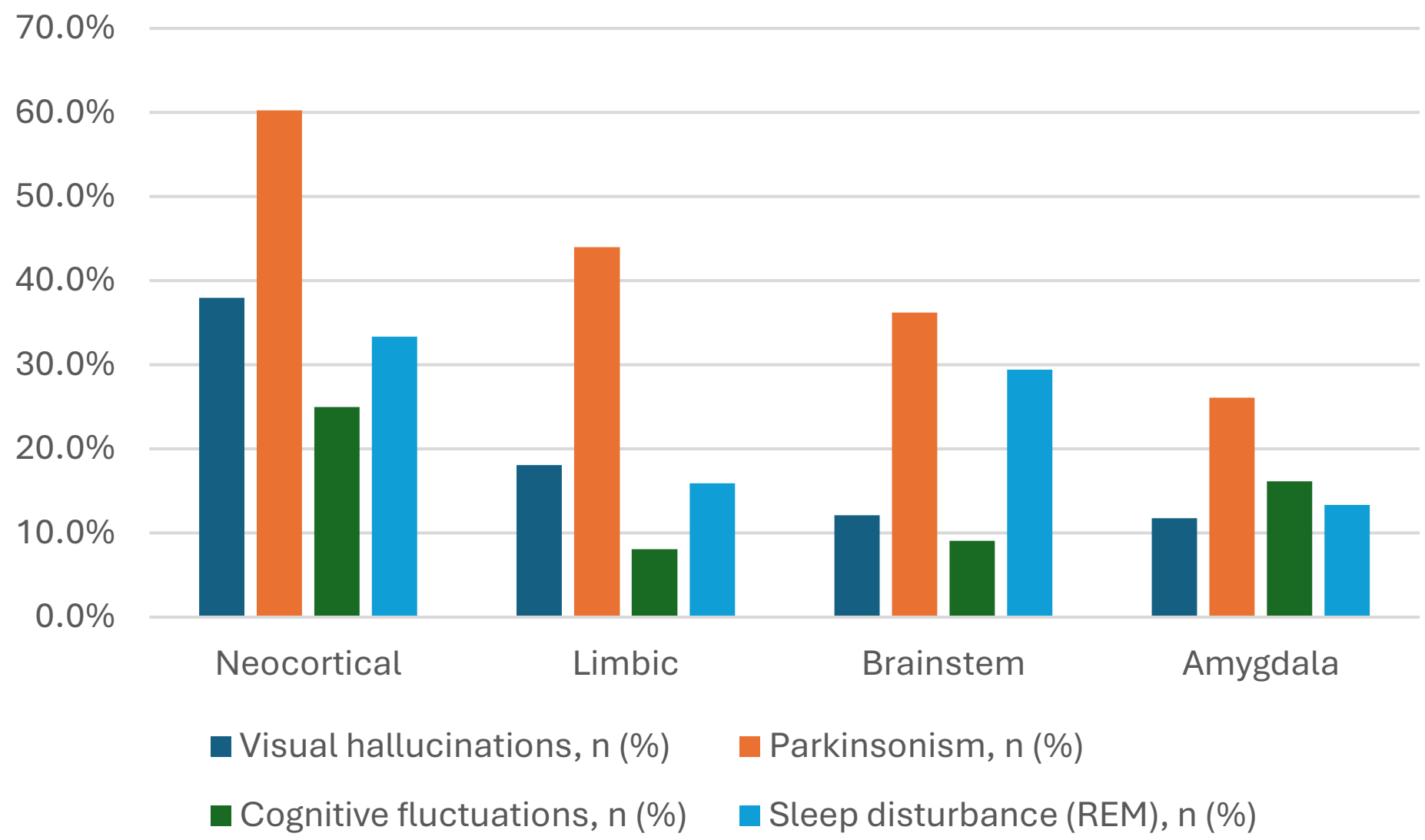

References

1. Kukull WA, Higdon R, Bowen JD, et al. Dementia and Alzheimer disease incidence: a prospective cohort study. *Arch Neurol*. Nov 2002;59(11):1737-46. doi:noc20207 [pii]
2. McKeith IG, Dickson DW, Lowe J, et al. Diagnosis and management of dementia with Lewy bodies: third report of the DLB Consortium. *Neurology*. Dec 27 2005;65(12):1863-72. doi:10.1212/01.wnl.0000187889.17253.b1
3. Suemoto CK, Leite REP, Ferretti-Rebustini REL, et al. Neuropathological lesions in the very old: results from a large Brazilian autopsy study. *Brain Pathol*. Nov 2019;29(6):771-781. doi:10.1111/bpa.12719
4. Suemoto CK, Ferretti-Rebustini RE, Rodriguez RD, et al. Neuropathological diagnoses and clinical correlates in older adults in Brazil: A cross-sectional study. *PLoS Med*. Mar 2017;14(3):e1002267. doi:10.1371/journal.pmed.1002267
5. Grinberg LT, Ferretti RE, Farfel JM, et al. Brain bank of the Brazilian aging brain study group - a milestone reached and more than 1,600 collected brains. *Cell Tissue Bank*. 2007;8(2):151-62. doi:10.1007/s10561-006-9022-z
6. Ferretti REL, Damin AE, Brucki SMD, et al. Post-Mortem diagnosis of dementia by informant interview. *Dement Neuropsychol*. Apr-Jun 2010;4(2):138-144. doi:10.1590/S1980-57642010DN40200011
7. Morris JC. The Clinical Dementia Rating (CDR): current version and scoring rules. *Neurology*. Nov 1993;43(11):2412-4. doi:10.1212/wnl.43.11.2412-a
8. Mikolaenko I, Pletnikova O, Kawas CH, et al. Alpha-synuclein lesions in normal aging, Parkinson disease, and Alzheimer disease: evidence from the Baltimore Longitudinal Study of Aging (BLSA). *J Neuropathol Exp Neurol*. Feb 2005;64(2):156-62. doi:10.1093/jnen/64.2.156
9. O'Brien RJ, Resnick SM, Zonderman AB, et al. Neuropathologic studies of the Baltimore Longitudinal Study of Aging (BLSA). *J Alzheimers Dis*. 2009;18(3):665-75. doi:10.3233/JAD-2009-1179
10. Wang SJ, Guo Y, Ervin JF, Lusk JB, Luo S. Neuropathological associations of limbic-predominant age-related TDP-43 encephalopathy neuropathological change (LATE-NC) differ between the oldest-old and younger-old. *Acta Neuropathol*. Jul 2022;144(1):45-57. doi:10.1007/s00401-022-02432-5
11. Bennett DA, Schneider JA, Arvanitakis Z, Wilson RS. Overview and findings from the religious orders study. *Curr Alzheimer Res*. Jul 2012;9(6):628-45. doi:10.2174/156720512801322573
12. Bennett DA, Buchman AS, Boyle PA, Barnes LL, Wilson RS, Schneider JA. Religious Orders Study and Rush Memory and Aging Project. *J Alzheimers Dis*. 2018;64(s1):S161-S189. doi:10.3233/JAD-179939
13. McKeith IG, Galasko D, Kosaka K, et al. Consensus guidelines for the clinical and pathologic diagnosis of dementia with Lewy bodies (DLB): report of the consortium on DLB international workshop. *Neurology*. Nov 1996;47(5):1113-24. doi:10.1212/wnl.47.5.1113
14. Schneider JA, Arvanitakis Z, Yu L, Boyle PA, Leurgans SE, Bennett DA. Cognitive impairment, decline and fluctuations in older community-dwelling subjects with Lewy bodies. *Brain*. Oct 2012;135(Pt 10):3005-14. doi:10.1093/brain/aws234
15. McKhann G, Drachman D, Folstein M, Katzman R, Price D, Stadlan EM. Clinical diagnosis of Alzheimer's disease: report of the NINCDS-ADRDA Work Group under the auspices of Department of Health and Human Services Task Force on Alzheimer's Disease. *Neurology*. Jul 1984;34(7):939-44. doi:10.1212/wnl.34.7.939

16. McKeith IG, Perry EK, Perry RH. Report of the second dementia with Lewy body international workshop: diagnosis and treatment. Consortium on Dementia with Lewy Bodies. *Neurology*. Sep 22 1999;53(5):902-5. doi:10.1212/wnl.53.5.902
17. Roman GC, Tatemichi TK, Erkinjuntti T, et al. Vascular dementia: diagnostic criteria for research studies. Report of the NINDS-AIREN International Workshop. *Neurology*. Feb 1993;43(2):250-60. doi:10.1212/wnl.43.2.250
18. Kawas CH, Kim RC, Sonnen JA, Bullain SS, Trieu T, Corrada MM. Multiple pathologies are common and related to dementia in the oldest-old: The 90+ Study. *Neurology*. Aug 11 2015;85(6):535-42. doi:10.1212/WNL.0000000000001831
19. Corrada MM, Berlau DJ, Kawas CH. A population-based clinicopathological study in the oldest-old: the 90+ study. *Curr Alzheimer Res*. Jul 2012;9(6):709-17.
20. Melikyan ZA, Greenia DE, Corrada MM, Hester MM, Kawas CH, Grill JD. Recruiting the Oldest-old for Clinical Research. *Alzheimer Dis Assoc Disord*. Apr-Jun 2019;33(2):160-162. doi:10.1097/WAD.0000000000000260
21. Braak H, Del Tredici K, Rub U, de Vos RA, Jansen Steur EN, Braak E. Staging of brain pathology related to sporadic Parkinson's disease. *Neurobiol Aging*. Mar-Apr 2003;24(2):197-211. doi:10.1016/s0197-4580(02)00065-9
22. Schmitt FA, Nelson PT, Abner E, et al. University of Kentucky Sanders-Brown healthy brain aging volunteers: donor characteristics, procedures and neuropathology. *Curr Alzheimer Res*. Jul 2012;9(6):724-33. doi:10.2174/156720512801322591
23. Petersen RC, Smith GE, Waring SC, Ivnik RJ, Tangalos EG, Kokmen E. Mild cognitive impairment: clinical characterization and outcome. *Arch Neurol*. Mar 1999;56(3):303-8. doi:10.1001/archneur.56.3.303
24. Kero M, Raunio A, Polvikoski T, Tienari PJ, Paetau A, Myllykangas L. Hippocampal Sclerosis in the Oldest Old: A Finnish Population-Based Study. *J Alzheimers Dis*. 2018;63(1):263-272. doi:10.3233/JAD-171068
25. Forrest SL, Kovacs GG. Protein Pathologies in Oligodendroglia in Neurodegenerative Diseases. *Adv Neurobiol*. 2025;43:407-432. doi:10.1007/978-3-031-87919-7_14
26. Fischer P, Jungwirth S, Krampla W, et al. Vienna Transdanube Aging "VITA": study design, recruitment strategies and level of participation. *J Neural Transm Suppl*. 2002;(62):105-16. doi:10.1007/978-3-7091-6139-5_11
27. Kovacs GG, Milenkovic I, Wohrer A, et al. Non-Alzheimer neurodegenerative pathologies and their combinations are more frequent than commonly believed in the elderly brain: a community-based autopsy series. *Acta Neuropathol*. Sep 2013;126(3):365-84. doi:10.1007/s00401-013-1157-y
28. Harrison WT, Lusk JB, Liu B, et al. Limbic-predominant age-related TDP-43 encephalopathy neuropathological change (LATE-NC) is independently associated with dementia and strongly associated with arteriolosclerosis in the oldest-old. *Acta Neuropathol*. Aug 20 2021;doi:10.1007/s00401-021-02360-w
29. Schmitt FA, Nelson PT, Abner E, et al. University of Kentucky Sanders-Brown Healthy Brain Aging Volunteers: Donor Characteristics, Procedures, and Neuropathology. *Curr Alzheimer Res*. Apr 2 2012;9(6):724-33.
