# Supplementary material for: The prevalence of Lewy body pathology across nine international community-based cohorts
Source: Brain. Author manuscript; Available in PMC 2026 Oct 17. (PMC13675927; doi:10.1093/brain/awag112)
Supplement: Supplementary file 2 [file NIHMS2209851-supplement-Suppl_Table_1.docx]

**Supplementary Table 1.** Supplementary information on included cohorts

| **Cohorts (in alphabetical order)** | **Notes** | **Ref(s)** |
| --- | --- | --- |
| **Adult Changes in Thought (ACT)** | A population-based prospective cohort study investigating brain aging and dementia risk factors within Kaiser Permanente Washington (formerly Group Health Cooperative), an integrated health care delivery system in Washington State. Community-dwelling, adults aged 65 years and older without dementia are recruited from among Kaiser Permanente members residing in the Seattle area. The demographic composition reflects the surrounding county in which a majority of people are white and relatively well-educated. Consent for brain donation is not required at enrolment; however, brain autopsy consent is consistently obtained from approximately 25–30% of participants, with rates increasing in recent years. At study entry and every two years thereafter, participants undergo cognitive screening, physical function testing, medical history review, and assessments of functional status. | ^50^ |
| **Baltimore Longitudinal Study of Aging (BLSA)** | Longitudinal study of normal aging with subjects 65 years or older enrolled in a prospective autopsy program in Baltimore, Maryland. The study was initiated in 1958 with recruitment initially restricted to men; women were included from 1978. The autopsy program was established in 1986. The cohort is predominantly male, white, and highly educated. However, no clinical or demographic data accompanied the neuropathological data from the BLSA included in this study. | ^51,52^ |
| **Brazilian Biobank for Aging Studies (BAS)** | Population-based autopsy study with subjects who died from non-forensic causes of death in the city of Sao Paulo, Brazil. Participants were 50 years or older, with diverse ethnic composition similar to the overall death data from São Paulo. The study has been ongoing since 2003, and all the donations are sourced from the Autopsy Service of the University of Sao Paulo, the only service providing non-forensic autopsies (around 15,000 per year)  in this city of over 11 million inhabitants. In the first 10 years after it was established, 16% of the total number of deaths in the city were autopsied in the BAS. | ^53-55^ |
| **Duke-UNC Alzheimer’s Disease Research Center (Duke ADRC)** | Participants over 90 years of age at death were included in this study. Approximately 70% of the cohort had normal cognition at the time of recruitment. More than 95% of participants are White. Consensus meetings were held yearly to review and update the clinical diagnosis, based upon contemporaneous NIA-AA criteria. | ^56^ |
| **Rush Religious Orders Study – Memory and Aging Project (ROS-MAP)** | Both are Rush University ongoing epidemiologic longitudinal clinical-pathologic studies of older persons. The Religious Order Study (ROS) began in 1994 and recruits older priests, nuns, and brothers from 45 sites across the United States. The Rush Memory and Aging Project (MAP) began in 1997 and recruits participants from across the Chicagoland metropolitan area. In both studies, participants were enrolled at age 65 and above without known dementia and agreed to undergo annual clinical evaluation and interview, including detailed neuropsychological testing, and brain donation. One third had 12 or fewer years of education and 10% were members of an ethnic or racial minority group. | ^57,58^ |
| **The 90+ Study** | Community-based autopsy cohort in Southern California, USA. Participants in The 90+ Study were initially survivors of the Leisure World Cohort Study (LWCS), a longitudinal epidemiological investigation of a retirement community in Orange County, CA, established in the early 1980s. The 90+ Study began in 2003, when surviving LWCS participants aged 90 years and older as of January 1, 2003 were invited to enroll. Additional invitations were extended on January 1, 2008, and annually thereafter, to LWCS participants on reaching age 90. More recently, open recruitment of volunteers aged 90 and older outside of LWCS was initiated through multiple strategies including residential mailing lists, community talks, outreach to primary care physicians, local newspaper advertisements, and referrals relatives or friends of participants. LWCS participants were recruited irrespective of cognitive status, whereas non-LWCS volunteers were limited to those with no or mild dementia. Approximately two-thirds of participants are from the original LWCS, and one-third are non-LWCS volunteers. The cohort is predominantly White (98%) and 50% are college educated. | ^59,60^ |
| **U. Kentucky Alzheimer’s Disease Research Center (Uky ADRC)** | Community-based autopsy cohort in Lexington, Kentucky based on an NIH-funded ADRC. Recruitment began in the mid-1980’s, enrolling cognitively normal adults aged 70 years and older who were followed annually until death. Final diagnosis prior to death refers to the diagnosis of a consensus group including clinicians and pathologists, whereas the PDD or DLB clinical syndrome was evaluated by a neurologist during life. More than 95% of included autopsied participants were White/Caucasian. In 2012 the autopsy rate since inception was 75%, increasing to 85% for persons consented after 2005. Mean years of education in autopsied patients 16.0+2.5. | ^61^ |
| **Vantaa 85+** | A population-based study, which includes all the subjects who lived in the city of Vantaa, Finland, in 1991 and were 85 years or over. > 50% of the population were neuropathologically examined. | ^62^ |
| **Vienna Trans-Danube Aging (VITA)** | Community-based autopsy cohort in Vienna, Austria. The VITA study was established in 2000 and enrolled all residents of Vienna’s 21st and 22nd districts born between May 1925 and June 1926. Participants were recruited at age 75 and followed longitudinally. | ^9,63,64^ |
